# High-content image-based analysis and proteomic profiling identifies Tau phosphorylation inhibitors in a human iPSC-derived glutamatergic neuronal model of tauopathy

**DOI:** 10.1038/s41598-021-96227-5

**Published:** 2021-08-23

**Authors:** Chialin Cheng, Surya A. Reis, Emily T. Adams, Daniel M. Fass, Steven P. Angus, Timothy J. Stuhlmiller, Jared Richardson, Hailey Olafson, Eric T. Wang, Debasis Patnaik, Roberta L. Beauchamp, Danielle A. Feldman, M. Catarina Silva, Mriganka Sur, Gary L. Johnson, Vijaya Ramesh, Bruce L. Miller, Sally Temple, Kenneth S. Kosik, Bradford C. Dickerson, Stephen J. Haggarty

**Affiliations:** 1 Department of Neurology, Chemical Neurobiology Laboratory, Center for Genomic Medicine, Massachusetts General Hospital and Harvard Medical School, Boston, MA 02114 USA; 2 Department of Pharmacology, University of North Carolina School of Medicine, Chapel Hill, NC 27599 USA; 3 Department of Biochemistry & Molecular Biology, Center for NeuroGenetics, University of Florida, Gainesville, FL 32610 USA; 4 Department of Molecular Genetics & Microbiology, Center for NeuroGenetics, University of Florida, Gainesville, FL 32610 USA; 5 Center for Genomic Medicine, Massachusetts General Hospital and Harvard Medical School, Boston, MA 02114 USA; 6 Picower Institute for Learning and Memory, Massachusetts Institute of Technology, Cambridge, MA 02142 USA; 7 Department of Neurology, Memory and Aging Center, University of California, San Francisco, CA 94158 USA; 8 Neural Stem Cell Institute, Regenerative Research Foundation, Rensselaer, NY 12144 USA; 9 Department of Molecular, Cellular and Developmental Biology, Neuroscience Research Institute, University of California, Santa Barbara, CA 93106 USA; 10 Department of Neurology, MGH Frontotemporal Disorders Unit, Massachusetts General Hospital and Harvard Medical School, Charlestown, MA 02129 USA; 11 Present Address: Department of Pediatrics, Indiana University School of Medicine, Indianapolis, IN 46202 USA

**Keywords:** Chemical biology, Drug discovery, Neuroscience, Stem cells, Medical research, Molecular medicine

## Abstract

Mutations in *MAPT* (microtubule-associated protein tau) cause frontotemporal dementia (FTD). *MAPT* mutations are associated with abnormal tau phosphorylation levels and accumulation of misfolded tau protein that can propagate between neurons ultimately leading to cell death (tauopathy). Recently, a p.A152T tau variant was identified as a risk factor for FTD, Alzheimer's disease, and synucleinopathies. Here we used induced pluripotent stem cells (iPSC) from a patient carrying this p.A152T variant to create a robust, functional cellular assay system for probing pathophysiological tau accumulation and phosphorylation. Using stably transduced iPSC-derived neural progenitor cells engineered to enable inducible expression of the pro-neural transcription factor Neurogenin 2 (Ngn2), we generated disease-relevant, cortical-like glutamatergic neurons in a scalable, high-throughput screening compatible format. Utilizing automated confocal microscopy, and an advanced image-processing pipeline optimized for analysis of morphologically complex human neuronal cultures, we report quantitative, subcellular localization-specific effects of multiple kinase inhibitors on tau, including ones under clinical investigation not previously reported to affect tau phosphorylation. These results demonstrate the potential for using patient iPSC-derived ex vivo models of tauopathy as genetically accurate, disease-relevant systems to probe tau biochemistry and support the discovery of novel therapeutics for tauopathies.

## Introduction

Tauopathies encompass a wide range of neurodegenerative diseases including frontotemporal dementia (FTD) and Alzheimer's disease (AD). The age of symptom onset, which may include behavioural, cognitive, and/or motor impairments, typically occurs at about age 50 but can occur as early as the 20s or into the 70s and beyond^1^. A major goal in the field is to develop disease-modifying therapies that specifically target tau pathology^2,3^.

A characteristic feature of tauopathies is the intracellular misfolding and aggregation of the microtubule-associated protein tau into paired helical filaments and neurofibrillary tangles (NFT)^4,5^. In addition, pathological tau is also heavily post-translationally modified, for example by hyperphosphorylation: in the normal brain tau contains 2–3 moles of phosphate per mole of protein, but tau is 2- to 3-fold hyperphosphorylated in the AD brain^6^. Hyperphosphorylation of tau has been proposed to: (1) alter its interactions with other cytoskeletal proteins, thereby affecting microtubule dynamics within axons; (2) induce tau missorting from axons to the somatodendritic compartment which can cause synaptic dysfunction; (3) alter its degradation through the proteasome or through autophagy and its truncation by proteases; and (4) exacerbate tau aggregation^4^. These tau pathologies lead to neuronal dysfunction and death; excitatory neurons are especially vulnerable^7^. Most recently, the spread of tau between cells has been linked to hyperphosphorylated species that are taken up most efficiently by neurons^8^. These observations suggest that reducing the levels of tau, and in particular phosphorylated tau (p-Tau), could be a key tau-based therapeutic strategy, either by itself or in combination with other approaches, against neurodegeneration.

Tauopathies can be sporadic or familial. Since the first missense mutation in the tau gene, *MAPT*, was discovered more than two decades ago in inherited cases of FTD with parkinsonism linked to chromosome 17 (FTDP-17)^9–11^, greater than 40 pathogenic mutations in *MAPT* have been described in over 100 familial cases of FTD^12^. Recently, a rare variant of tau, p.A152T (alanine to threonine), located in the proline-rich domain upstream of the microtubule-binding domain, has been identified as a risk factor for FTD, AD and synucleinopathies^13–26^. This variant of tau has decreased affinity for binding microtubules in vitro^13^ and we have previously shown that induced pluripotent stem cells (iPSC)-derived neurons carrying this A152T variant (Tau-A152T) harbour a larger and more insoluble tau load than control neurons^27^. Furthermore, Tau-A152T neurons have increased sensitivity to exogenous cellular stressors; this increased sensitivity can be rescued by reducing tau protein levels, either pharmacologically or by gene-editing^27–29^. In the present study, we expand our work with iPSC from an FTD subject diagnosed with progressive supranuclear palsy carrying this A152T variant as a genetically accurate cell model of tauopathy, in order to enable screening for novel therapeutics. Using this model, we developed a rapid, reproducible, and scalable microwell neuronal differentiation system which consists of excitatory neurons generated by inducible expression of the pro-neural transcription factor Neurogenin 2 (Ngn2). We then utilized an image-based human neuronal cellular assay combined with high-content image analysis, capable of distinguishing tau levels in neuronal cell bodies and processes, to probe for compounds that can reduce total and p-Tau levels with an emphasis on clinically used FDA-approved drugs or drugs in clinical trial with potential for repurposing. This strategy will aid in expediting translational research in elucidating novel targets of therapeutic intervention for FTD-tau and other tauopathies^3^.

## Results

### Characterization of human iNgn2 neuronal cell model

In order to develop a high-content imaging assay useful for small-molecule screening and functional studies, we sought to develop a neuronal cellular system that was physiologically relevant while also rapid, reproducible and scalable. We reasoned that ideal physiological relevance could be achieved with the use of human neurons derived from both FTD patient and healthy control iPSC. In particular, using excitatory human neurons seemed appropriate given the special vulnerability of these neurons to dysfunction and death cause by tauopathy^7^. Lastly, for reproducibility and scalability, we needed to engineer stable cell lines that could be rapidly differentiated into neurons in microwell plates.

**Figure 1:**
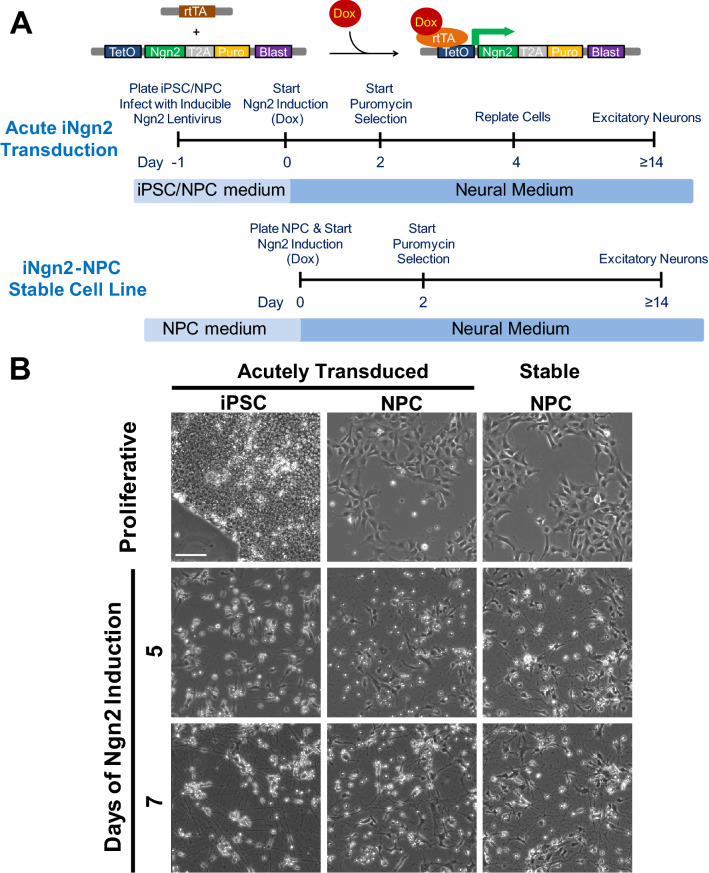
Schematic of the methodology and morphology of iNgn2 neurons. (**A**) Top panel illustrates the action of the transgenes in inducing the expression of Ngn2 in generating the iNgn2 neurons. The addition of doxycycline (Dox) results in the binding of Dox to the transactivator (rtTA) expressed of a constitutively active human ubiquitin C promoter, changing the conformation of rtTA to promote binding of the rtTA-Dox complex to the inducible TetO promoter, thereby activating the expression of Ngn2 and the puromycin (Puro) resistance marker. Stable cell lines were generated before neuronal induction with the use of the constitutively expressed blasticidin (Blast) resistance selective marker. The bottom two panels contain the timeline of iNgn2 neuron generation either by acute transduction of the Ngn2 lentivirus or induction of Ngn2 in an iNgn2-NPC stable cell line. Human iPSC or NPC were plated and transduced with the Ngn2 lentivirus at day -1 in the acute transduction method. Neuronal induction was started at Day 0 with the addition of Dox. Transduced cells were selected at Day 2 with the addition of Puro. Optionally, puro-selected iNgn2 cells can be replated at a desired density at Day 4. By Day 14, predominantly excitatory glutamatergic neurons are generated. (**B**) Phase contrast images of live iNgn2 neurons at various time points of Ngn2 induction. Scale bar represents 100 μm.

Recently, Zhang et al.^30^ described a method of differentiation of iPSC into functional, predominantly excitatory, cortical-like, glutamatergic human neurons in approximately 14 days that involves inducible expression of the pro-neural transcription factor Neurogenin 2 (iNgn2). While the speed of obtaining iPSC-derived neurons using iNgn2 is a highly favourable trait for a neuronal cell-based screening assay, the original protocol described^30^ contains more steps than is ideal for high-throughput application, and additional applications while demonstrating the promise of this technique of Ngn2 induction in the context of tau phenotypes have generally worked directly from iPSCs lines which has limit on the scalabilty^31–33^. Thus, we set out to streamline the cell handling steps of the iNgn2 neuronal system with the objective of developing neuronal high-content imaging assays in cortical-like, glutamatergic neurons (Fig. 1A). To begin our optimization, we first used a custom neural media (N3aM^34^) that was introduced to the cells at day 0, at the same time as the start of iNgn2 expression with the addition of doxycycline. We did not add glia to the neuronal culture; instead we tested the effect of feeding cells with or without astrocyte-conditioned media (ACM; Supplementary Fig. S1). By 14 days of iNgn2, there was robust total tau (TAU5) and p-Tau (Tau-pS396) protein expression detectable in the neurons, either with or without ACM treatment, whereas expression of the synaptic proteins PSD-95 and Synapsin 1 was either similar or only slightly more enhanced by ACM relative to N3aM alone. In addition, expression of tau and synaptic proteins was greater when the neurons were fed with fresh media containing brain-derived neurotrophic factor (BDNF), neurotrophin-3 (NT3) and doxycycline (GF + dox) every other day (Supplementary Figs. S1 and S2). Thus, for the cell-based screening assay, the cells were fed every other day with fresh N3aM, and ACM was omitted from the protocol.

**Figure 2:**
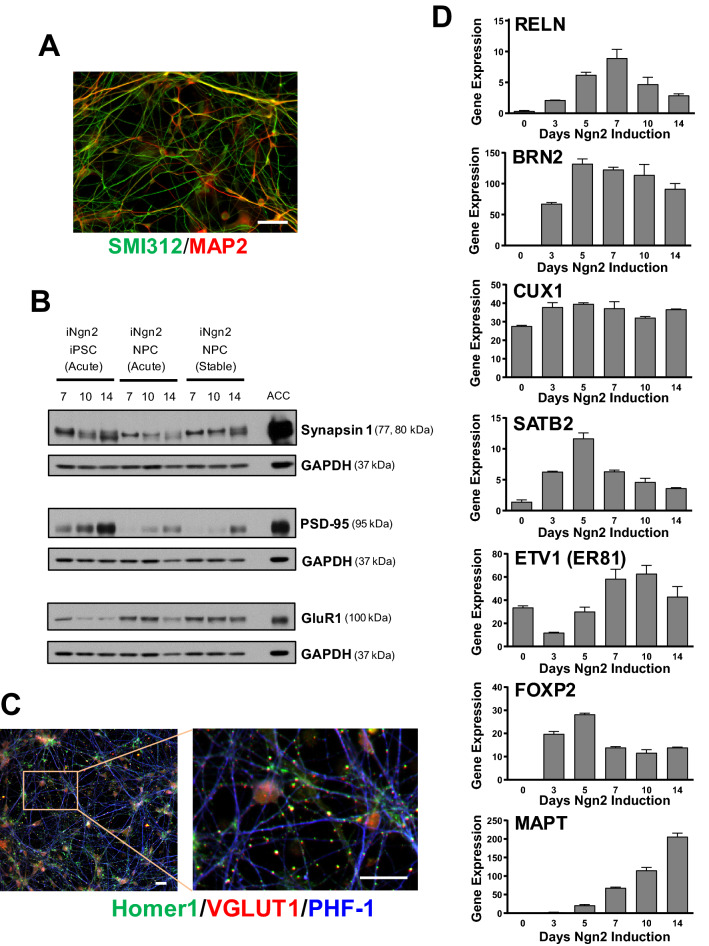
Expression of cortical glutamatergic excitatory markers and tau in iNgn2 neurons. (**A**) Immunostaining of SMI312 (axonal marker) and MAP2 (dendritic marker) in 10-day iNgn2 neurons. Scale bar represents 50 μm. (**B**) As assessed by western blots, there is robust expression of synaptic proteins Synapsin 1 and PSD-95, and the glutamatergic marker GluR1 in 14-day iNgn2 neurons from acutely transduced iPSC and NPC, and the iNgn2-NPC stable cell line. For comparison, human brain tissue from the anterior cingulate cortex (ACC) was also probed. Full-length blots are presented in Supplementary Fig. S11. (**C**) By 18 days of iNgn2, the neurons exhibit numerous synaptic punctae consisting of colocalized presynaptic marker VGLUT1 and postsynaptic marker HOMER1 along neuronal processes immunostained with the p-Tau marker PHF-1. Scale bar represents 20 μm. (**D**) These neurons also express cortical layer marker and tau mRNA (as transcripts per million, TPM) as determined by RNAseq.

Using this culturing protocol, we were able to rapidly generate neurons from either iPSC or neural progenitor cells (NPC), with robust neurite outgrowth detectable as early as day 4–5 (Fig. 1B), elongated neurite extension by day 7, and an extensive meshwork of neurons by day 10–14. At this stage (day 10 of iNgn2), this meshwork of neurons contains clearly separated axonal and dendritic processes, henceforth interchangeably referred to as neuronal processes or neurites (Fig. 2A). By day 14, iNgn2 neurons express the synaptic proteins Synapsin 1, PSD-95 and the glutamatergic neuron marker GluR1 (AMPA receptor 1), as observed by western blot (Fig. 2B). Moreover, PHF-1, recognizing an epitope around S396 and S404 phosphorylated sites (p-Tau)^35^, staining could be detected in neuronal processes with adjacent glutamatergic (VGLUT1) synaptic (Synapsin 1) punctae using immunofluorescence imaging as early as day 10 of iNgn2 (Supplementary Fig. S3). By day 18, there was extensive presynaptic (VGLUT1) and postsynaptic (Homer 1) punctae colocalization (Fig. 2C). To complement the single-cell level imaging analysis, we examined the expression of cortical and subcortical markers in iNgn2 neurons at various time points using RNAseq. In general, iNgn2 neurons expressed genes for each of the cortical and subcortical layers (Fig. 2D), with the highest level of expression of marker for the upper cortical layers II-III (*BRN2/POU3F2*). This high level of expression of BRN2 during two weeks of neuronal differentiation, as compared to iPSC, has also been reported by others^36^. The expression of other cortical markers, such as CUX1 and ETV1, in iPSC has also been reported by others^36^, however their expression levels increase further during Ngn2-mediated neuronal differentiation. *MAPT* mRNA, consisting exclusively of 0N3R transcripts, was detectable by day 5, and increased thereafter (Fig. 2D). These results indicate that with iNgn2, we are generating predominantly glutamatergic excitatory neurons with upper cortical layer features, which is consistent with the results shown in the original report of this method^30^.

**Figure 3:**
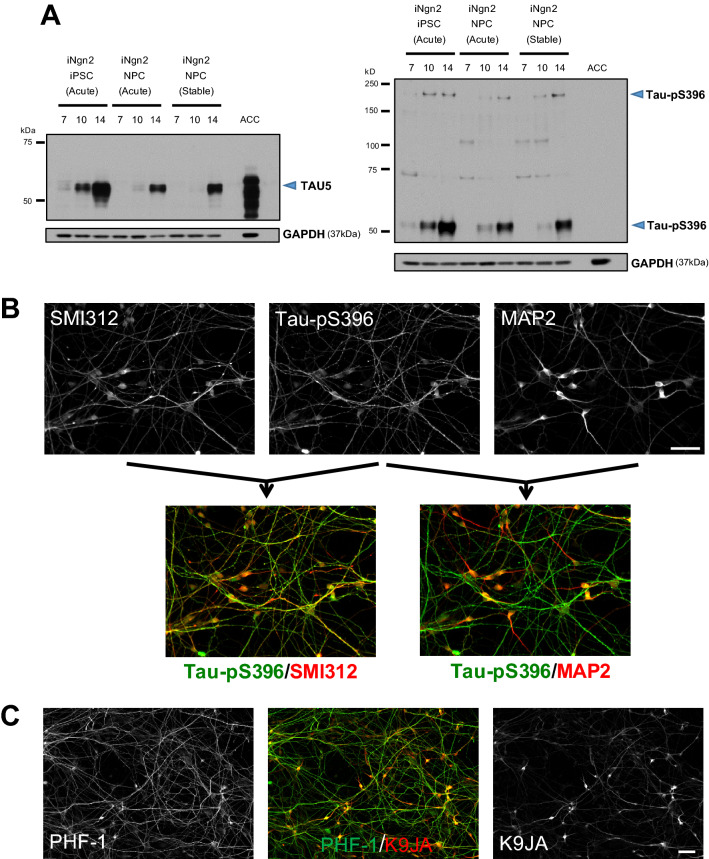
Protein expression and morphological characteristics of tau in iNgn2 neurons. **(A)** As assessed by western blots, there is robust expression of total tau (TAU5) and p-Tau (Tau-pS396) proteins in 14-day iNgn2 neurons from acutely transduced iPSC and NPC, and the iNgn2-NPC stable cell line. For comparison, human brain tissue from the anterior cingulate cortex (ACC) was also probed for tau expression. p-Tau was not detected in the ACC sample. This could be due to either enhanced tau phosphorylation in our iNgn2 neurons, or artifactual dephosphorylation that occurred during processing of the autopsied brain^38^. Full-length blots are presented in Supplementary Fig. S11. (**C**) Morphologically assessed by immunofluorescence imaging, p-Tau (Tau-pS396) preferentially colocalizes with the axonal marker (SMI312) and not the dendritic marker (MAP2) in 14-day iNgn2 neurons. Scale bar represents 50 μm. (**D**) Immunostaining of p-Tau (PHF-1) and total tau (K9JA) in 14-day iNgn2 neurons. Scale bar represents 50 μm.

iNgn2 differentiation of iPSC or NPC resulted in the emergence of total and p-Tau (Tau-pS396), with robust expression by day 14 (Fig. 3A, control neurons; Supplementary Fig. S4, Tau-A152T neurons) in both control and Tau-A152T neurons. Co-staining with axonal (SMI312) and dendritic (MAP2) markers indicated that p-Tau was preferentially localized in axons in these neurons (Fig. 3B), with low levels of p-Tau (with either PHF-1, Tau-pS396 or AT8 antibodies) and total tau (K9JA) immunostaining in the cell body and all other neuronal processes (Fig. 3C and Supplementary Fig. S3). The preferential axonal localization of tau is consistent with what is seen in other neuronal cell culture systems with this polarization^37^, an important measure of maturation.

Next, to further streamline the cell culture methodology required for scalable generation of large quantities of iNgn2 neurons, we made iNgn2-containing NPC (iNgn2-NPC) stable cell lines by introducing a constitutively expressed blasticidin resistance gene into the original rtTA-driven Tet-O-Ngn2 lentivirus construct, and then transducing and selecting NPC which had stably incorporated this construct. We made these stable iNgn2-NPC because culturing iPSC at a large scale needed for a high content screening assay would be impractical due to the high cost of the media and the labour intensive nature of the culture process. Similarly, transduction of NPC by infection with lentiviruses containing the inducible Ngn2 expression cassette for every assay run, would be too cumbersome and time-consuming. Thus, the use of NPC with stably incorporated inducible Ngn2 expression cassette, and induction of neuronal differentiation by treatment of cells with doxycycline, represents the most practical way to generate neurons at high scale in multi-well plates suitable for a high content screening assay. With these iNgn2-NPC stable cell lines, we were able to further reduce the number of cell culture days required to generate neurons by seeding the cells and inducing Ngn2 expression on the same day (day 0; see Fig. 1), and then feeding the emerging neurons on subsequent days. The iNgn2 neurons produced either by acute transduction of iNgn2 lentivirus into NPC or iNgn2-NPC stable cells appear to develop into neurons with similar properties when assessed morphologically (Fig. 1B) or by probing for expression of tau, synaptic or glutamatergic markers (Figs. 2 and 3). In summary, we have optimized a human neuronal cell culture system for the large-scale, directed differentiation of cortical-like, excitatory, glutamatergic neurons that is amenable for use in high-content screening assays.

### Optimization of multiplexed assay parameters

**Figure 4:**
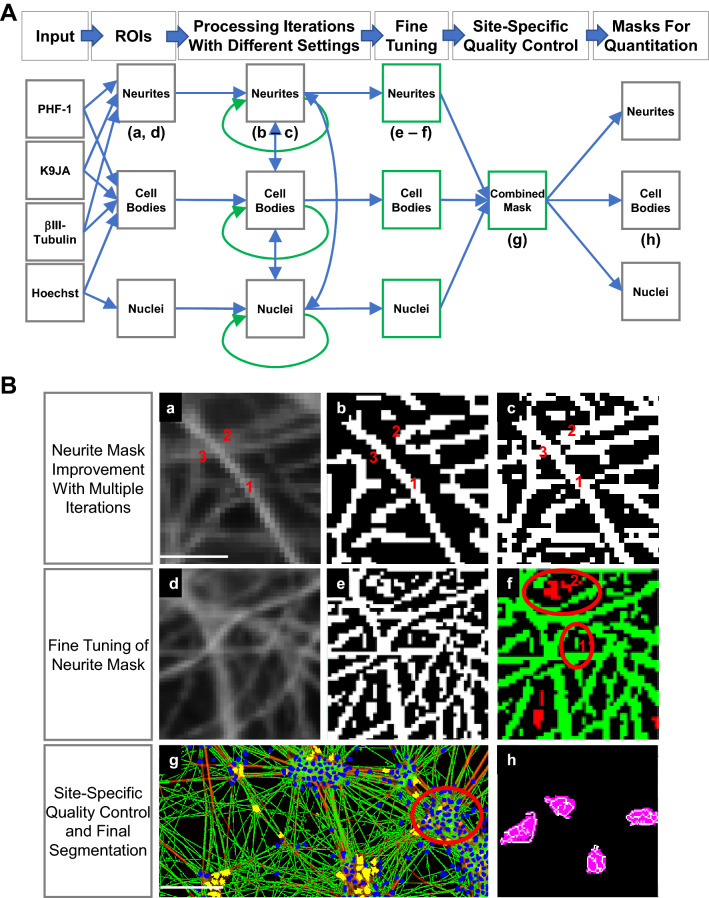
Image segmentation pipeline. (**A**) Schematic diagram of processing steps in the image segmentation pipeline. Information derived from different combinations of imaged channels is used as initial input for segmentation of 3 different subcellular regions of interest (ROI). For segmentation, multiple iterations of a multi-part process are successively used to extract ROI-specific masks; information from each ROI is used to inform identification of the other ROIs; post-iterative extraction, ROIs undergo further fine tuning processes; the combined masks are subject to additional quality control to provide final masks for quantification. Blue arrows indicate information input direction; green arrows/squares indicate processing steps involved, letters reference insets in B. (**B**) Examples highlighting different steps within the image segmentation pipeline. (**a**–**c**) Comparison of 1st (**b**) vs final iteration (**c**) of neurite mask extraction for input example (**a**) highlighting the advantages of multi-part multi-iteration process: improved segmentation for both major (1) and minor (2) neurites; (3) improved neurite path. (**d**–**f**) Example of fine tuning for neurite mask. Input (**d**) leads to post-iterative extraction of neurite mask (**e**): neurite segments undergo test to differentiate between true neurite segments (green, 1) and background signal (red, 2). (**g**,**h**) Final quality control steps. Combined segmentation mask (**g**): neurites (green), neuronal cell bodies (yellow), non-neuronal nuclei (blue). Cell bodies and neurite sections where clumps occur (**g**, red circle) are excluded from quantification. Neuronal cell body-derived quantification is restricted to areas NOT crossed by neurites (**h**, white lines). Scale bars represent 10 µm for (**a**–**f**,**h**). Scale bar represents 100 µm in (**g**).

Having established a robust and scalable culture system to obtain cortical-like, glutamatergic neurons in a 96-well plate format, we turned next to optimizing a multiplexed, imaging-based assay by performing co-immunostaining/small molecule staining: (1) total tau with an antibody that recognizes all isoforms of tau (K9JA); (2) p-Tau with the PHF-1 antibody that recognizes phosphorylation on residues S396 and S404 and identifies hyperphosphorylated tau in NFT in patient brain tissue^35,39^; (3) neuronal microtubule cytoskeleton with an antibody to βIII-tubulin; and (4) DNA with Hoechst 33342. After automated imaging of the fluorescence signals of the four channels, the images were processed through a custom segmentation and data analysis pipeline to quantify total and p-Tau in neuronal processes and cell bodies (Fig. 4). Given the intrinsic morphologically complex iNgn2 neurite meshwork (e.g. Fig. 4 and Supplementary Fig. S5), we implemented a fully automated image segmentation pipeline able to identify and separately measure fluorescence signals in neuronal processes and cell bodies using the IN Cell Developer Toolbox 1.9.2. The segmentation pipeline consists of four main parts (Fig. 4). First, by successively detecting localized shifts in signal intensity and correspondingly applying different cut-offs for the involved parameters, nuclei, neuronal cell bodies, and neuronal processes are identified. Next, a number of fine-tuning steps distinguishing minor (comparatively dim, thin) neurites from background signal and non-random gap closing, enhance neurite detection. Having identified the initial cell body and neurite segmentation masks, site-specific quality control procedures are implemented, including exclusion of cell clumps or antibody-dependent background. In addition, for cell body-derived measures, crossing neurite regions are excluded. Finally, fluorescence signals in cell bodies and neurites identified by this process are quantitated. Throughout the entire segmentation pipeline, we have geared the stringency of the settings towards reduction of false positives rather than false negatives. Owing to the robustness of the iterative segmentation approach, the final pipeline requires not more than 3 assay run-to-assay run-specific adjustments to compensate for potential variation in global signal intensity due to technical variability, for example different antibody aliquots or instrument variation. Using this approach, we were able to successfully segment both sparse and dense neuronal process networks, and distinguish between major neuronal processes (comparatively bright, thick) and minor neurites (comparatively dim, thin) using the same set of parameters and settings. In addition, the sum of the total area of all neurites was used as a measure of cell health; small molecules producing substantial reductions in total neurite area could be deemed neurotoxic.

We validated our custom analysis pipeline by quantitating levels of tau and p-Tau in our iNgn2 neurons, and quantitating the effect of a positive control small molecule (CHIR-99021) known to decrease tau (Supplementary Fig. S6). As expected, over a time course of 14 days of iNgn2 differentiation, there was a gradual increase in neurite area (measured using βIII-tubulin); an increase in neurite area was also detected upon increasing cell plating densities (Supplementary Fig. S6). Similarly, there was an increase in both p-Tau (PHF-1) and total tau (K9JA) mean intensity in both cell body and neuronal processes with both increasing cell density and time in culture, with the total tau levels in the cell body plateauing earlier and with lower cell densities in the cell body than p-Tau, indicating that tau phosphorylation continues to increase over time in culture even after the total level of tau has reached a plateau. Finally, to analyse the effects of a small molecule on the levels of tau expression and tau phosphorylation in iNgn2 neurons, we sought to validate a potent and selective inhibitor of tau phosphorylation. Based upon our previous studies in mouse neurons and human NPC assays of WNT/GSK3 signaling^40,41^, we selected CHIR-99021 to measure the sensitivity of the assay to detect PHF-1 intensity changes in the cell body and neuronal processes. Consistent with the notion that PHF-1 is specific to the phospho-epitope S396/S404 generated at least in part by GSK3 kinase activity^42^, CHIR-99021 (10 µM) treatment decreased tau phosphorylation in 14-day iNgn2 neurons (Supplementary Fig. S6). These effects of CHIR-99021 were most pronounced at higher cell density. Thus, in subsequent experiments, the iNgn2-NPC stable cells were plated at an optimal cell density of at least 2.5 × 10^4^ cells/well in the 96-well plate format and differentiated for 13–14-day before cultures were treated with small molecules. Taken together, these results indicate that our custom analysis pipeline functions as expected to quantitate tau and p-Tau levels in differentiating human neurons, and highlight the importance of carefully considering both the time period of neuronal differentiation and the impact of factors that influence cell density when assaying tau phosphorylation.

### Chemoproteomic analysis of the selectivity of CHIR-99021 toward the kinome in iNgn2 glutamatergic neurons

**Figure 5:**
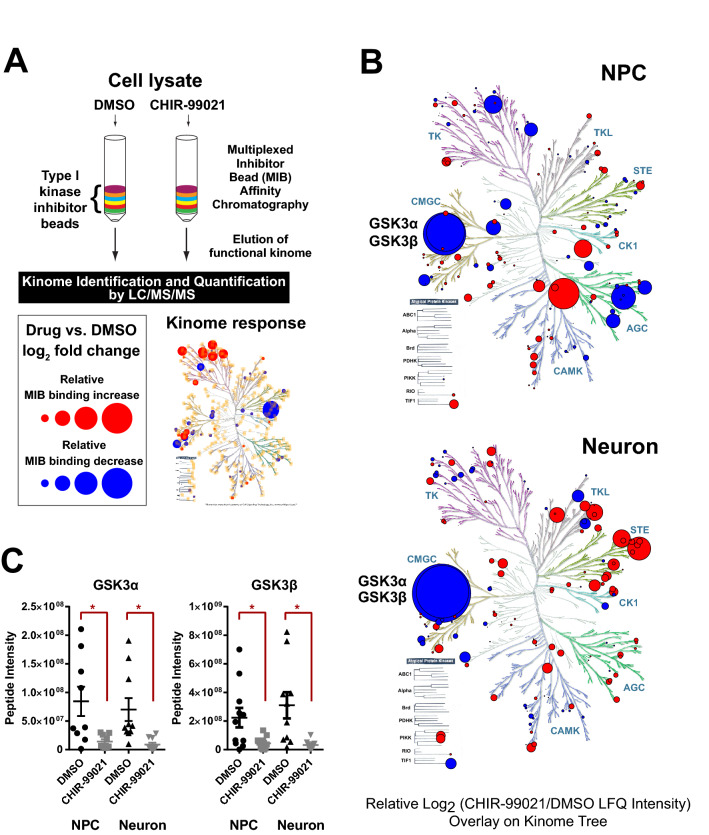
Effect of CHIR-99021 on the kinome in human NPC and neurons. (**A**) Schematic of the MIB-MS (Multiplexed Inhibitor Bead matrix followed by Mass Spectrometry) methodology. (**B**) Response of kinases in NPC and iNgn2 neurons to 24 h treatment with CHIR-99021 (1 μM) represented in a kinome tree. Magnitude of the LFQ intensity is represented by the sizes of the dots in the kinome tree; blue dots represent negative values and red dots represent positive values. (**C**) Scatter dot plot of GSK3α and GSK3β peptide intensity from mass spectrometry in NPC or neurons with CHIR-99021 (1 μM) or DMSO treatment. For each condition, the horizontal line represents the mean and the error bar represents SEM. Unpaired t-test with Welch’s correction was performed, *p < 0.05. Kinome trees produced using Kinome Render and illustration reproduced courtesy of Cell Signaling Technology, Inc. (www.cellsignal.com)^80^.

The lowering of PHF-1 immunostaining produced by CHIR-99021 in our neuronal cell model seemed most likely due to its inhibition of GSK3, given its high selectivity for GSK3 over related kinases determined with in vitro assays^43^. However, we reasoned that it was important to further validate its selectivity in our cells since: (i) the selectivity of CHIR-99021 towards GSK3 in human iPSC-derived neurons had never been determined; (ii) it was unclear whether CHIR-99021 had been tested for inhibition of kinases expressed in cortical, glutamatergic neurons; and (iii) both MAPK and CDK5 have been reported to also phosphorylate tau at S396/S404 in cells^44^, and thus, in principal, CHIR-99021 could have lowered PHF-1 staining through inhibition of kinases other than GSK3. To address these issues, and to more generally characterize the kinome for the first time in iNgn2 neurons, we used a chemoproteomic approach using a Multiplexed Inhibitor Bead Matrix-Mass Spectrometry assay (MIB-MS)^45–47^. MIB-MS utilizes immobilized Type I kinase inhibitors to selectively enrich the kinases from cell lysates based on their activity, expression level, and affinity for the inhibitor beads. Bound kinases are eluted, subjected to MS analysis and quantified (Fig. 5A). Decreased binding of a kinase in cell lysates flowed over the column after cell treatment with a test compound indicates binding of the test compound to the kinase and thus occlusion of binding to immobilized inhibitors on the column. Using this MIB-MS assay, the binding of CHIR-99021 to the kinome in both NPC and iNgn2 neurons was determined. Treatment of either human NPC or neurons with CHIR-99021 (1 μM) for 24 h resulted in ~ 3 and ~ 2 log_2_ CHIR-99021/DMSO fold decreases in GSK3 binding in neurons and NPC, respectively (Fig. 5B). Binding levels of other kinases, either within the CMGC (Cyclin-dependent kinase, e.g. CDK5; Mitogen-activated protein kinase, e.g. MAPK; Glycogen synthase kinase, CDC-like kinase) or other kinase families, were minimally affected (specific kinases and values are provided in Supplementary Fig. S7). This effect of CHIR-99021 was similar between the two GSK isoforms, GSK3α and GSK3β (Fig. 5C) in both NPC and neurons. Thus, we concluded that CHIR-99021 is a highly selective GSK3 inhibitor in human NPC and neurons and therefore suitable for use as positive control in our assays of tau phosphorylation.

### Comparison of iNgn2 neurons from a healthy control versus Tau-A152T patient

Having developed a culture system to generate cortical-like, glutamatergic neurons, we sought next to compare the resulting tau biochemical profile between healthy control neurons versus neurons from an A152T tau variant carrier. Similar to our published studies in which neurons were differentiated by a different method (growth factor withdrawal)^27^, we again consistently observed increased p-Tau and total tau in Tau-A152T neurons compared to controls by western blot (also see Fig. 7A and Supplementary Fig. S8). A quantitative comparison of the subcellular localization of tau between control and Tau-A152T neurons consistently shows higher p-Tau and total tau in the cell body of the Tau-A152T neurons (the neuronal processes/cell body ratio is lower) relative to control neurons (Supplementary Table S1). One of the pathological phenotypes in tauopathy, both in transgenic animal models and in human brain, is the redistribution of tau in neurons from the axons to the cell body and dendrites^48^. Therefore, our high-content tau imaging assay has the capability to not only detect compounds that alter tau phosphorylation and levels in the whole cell, but also has the potential to detect compounds that reduce pathologically-relevant somatodendritic tau redistribution.

### Image-based analysis of neuronal p-Tau and total levels and localization after treatment of cultures with small molecules

**Figure 6:**
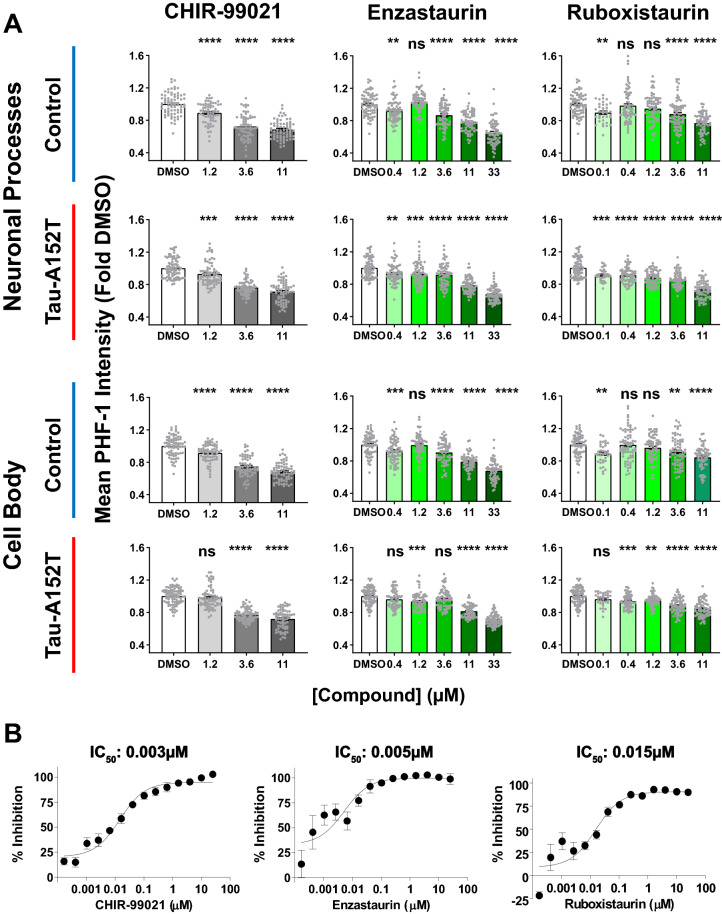
Dose-dependent effect of selected compounds on p-Tau in human neurons and in an in vitro GSK3β assay. (**A**) 20 h treatment with CHIR-99021, enzastaurin and ruboxistaurin dose-dependently decreased mean PHF-1 intensity in both cell bodies and neuronal processes in both control and patient iNgn2 neurons. Mean ± SEM of two biological replicates; dots represents individual values. One-way ANOVA, Dunnett’s post hoc test, ns = not significant, *p ≤ 0.05, **p ≤ 0.01, ***p ≤ 0.001, ****p ≤ 0.0001 compared to DMSO. (**B**) IC_50_ values of CHIR-99021 (positive control, n = 2), enzastaurin (n = 4) and ruboxistaurin (n = 4) as determined in the ADP-Glo kinase assay for GSK3β, where n is the number of replicate wells.

Having validated CHIR-99021 as a highly selective GSK3 inhibitor in iNgn2 neurons and demonstrated phenotypic differences in tau comparing healthy control to Tau-A152T neurons, we sought to determine CHIR-99021 potency toward lowering PHF-1 immunoreactivity. We also used the ability of our high-content imaging assay to sensitively quantify subcellular levels of tau expression and its phosphorylation status using antibody co-staining to measure total tau levels (K9JA) and βIII-tubulin as a general neuronal marker. CHIR-99021 showed a dose-dependent effect on mean PHF-1 intensity (Fig. 6A) lowering the intensity of staining both in neuronal processes and cell bodies of control and Tau-A152T neurons. The z-factor for mean PHF-1 intensity in control neurons was 0.5 in the cell body and 0.4 in the neuronal processes (in patient neurons, 0.4 in cell body and 0 in neuronal processes). Similarly, CHIR-99021 also caused a dose-dependent decrease of the ratio of PHF-1/K9JA (p-Tau/total tau) mean intensities (Supplementary Fig. S9) in both neuronal processes and cell body of control and Tau-A152T neurons. The z-factor for the PHF-1/K9JA mean intensity ratio in control neurons was 0.6 in the cell body and 0.4 in the neuronal processes (in patient neurons, 0.4 in cell body and 0.3 in neuronal processes). Importantly, CHIR-99021 had no effect on total neurite area as measured by βIII-tubulin staining, indicating that its lowering of tau and p-Tau levels was not due to neurotoxicity in our cultures.

**Figure 7:**
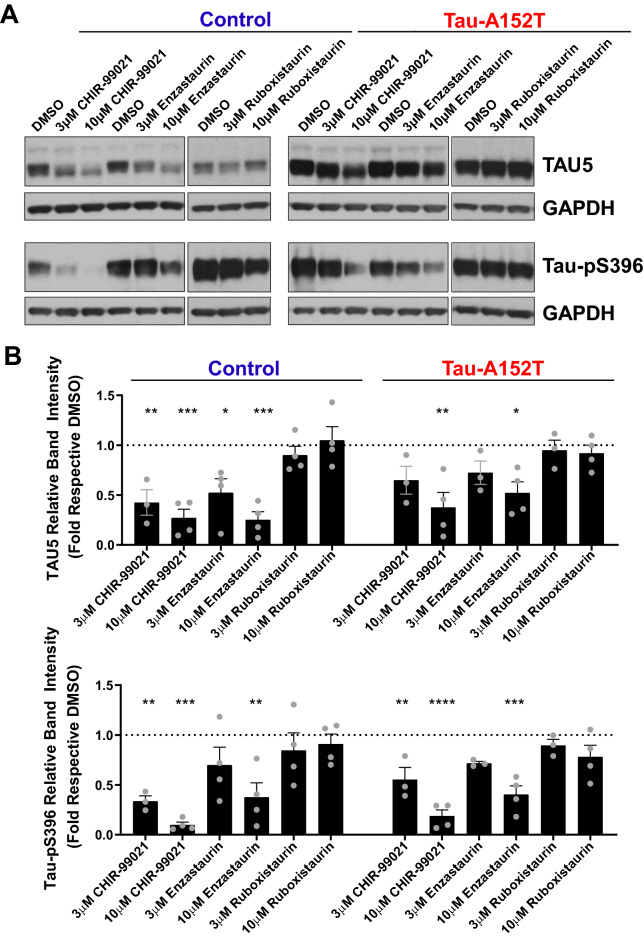
Effect of compounds on tau protein levels. Representative western blot images (**A**) from one of 3–4 replicate sets collected per compound. Full-length blots are presented in Supplementary Fig. S12. (**B**) Quantitation of western blot band intensities showing the effect of 20 h treatment with CHIR-99021, enzastaurin or ruboxistaurin on TAU5 and Tau-pS396 in both control and Tau-A152T iNgn2 neurons. Each bar represents mean ± SEM of at least 3 biological replicates; dots represent individual values. TAU5 and Tau-pS396 band intensities were normalized to GAPDH band intensities to control for variation in sample loading. To account for blot to blot variability in signal intensity, band intensities for drug treatment lanes were normalized to the DMSO band intensity of each cell line in each blot. The dotted line represents the GAPDH-normalized signal intensity of DMSO, set to 1. One-way ANOVA, Dunnett’s post hoc test, *p ≤ 0.05, **p ≤ 0.01, ***p ≤ 0.001, ****p ≤ 0.0001 compared to DMSO.

We also tested the effect of two multi-kinase inhibitors, enzastaurin and ruboxistaurin, in our neuronal cell-based imaging assay. These two compounds are most commonly considered as PKC inhibitors, but they have been shown to bind to multiple other kinases, with binding affinities for members of the AGC family (which includes PKC) and CMGC family (which includes CDKs and GSKs) in the low nanomolar range^49,50^. In particular, using an in vitro enzymatic assay, we found that enzastaurin and ruboxistaurin inhibited GSK3β with IC_50_ of 5 nM and 15 nM, respectively (Fig. 6B). Consistent with this potent inhibition of GSK3β, in the high-content imaging assay, both enzastaurin and ruboxistaurin dose-dependently decreased mean PHF-1 intensity in both neuronal processes and cell bodies of control and Tau-A152T neurons (Fig. 6). Interestingly, the two compounds were found to differ in their effect on the PHF-1/K9JA mean intensity ratio: enzastaurin had a more significant dose-dependent effect, whereas ruboxistaurin had a more modest effect mostly in the cell body at concentrations greater than 3 µM (Supplementary Fig. S9). Analysis of total tau (using TAU5 antibody) and p-Tau (using Tau-pS396 antibody) western blot analysis corroborated the imaging data with both CHIR-99021 and enzastaurin showing a dose-dependent decrease in total tau and p-Tau levels in both control and Tau-A152T neurons, whereas ruboxistaurin’s effect was more limited (Fig. 7). These results may be due to the more potent inhibitor effect of enzastaurin on GSK3 kinase or alternatively additional targets that also contribute to tau phosphorylation and control of tau levels. Importantly, the observation of elevated total tau and p-Tau levels in Tau-A152T neurons versus controls (Fig. 7) are in agreement with our previous observations in using NPC-derived neurons through growth factor withdrawal^27^.

**Figure 8:**
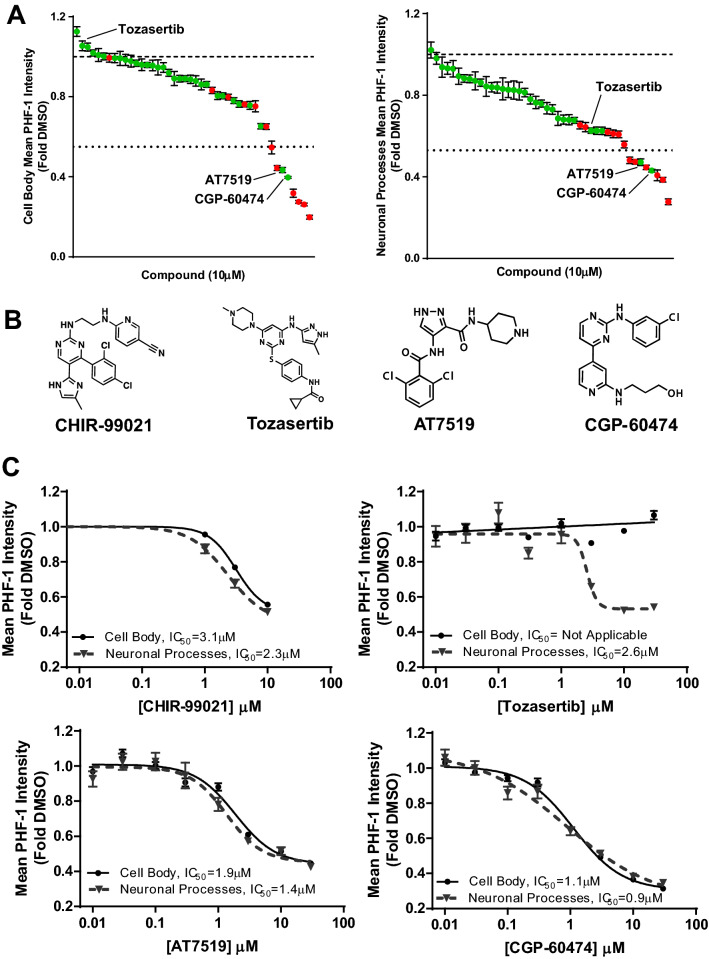
Effect of compounds from a custom collection of 44 small molecule kinase inhibitors on p-Tau (PHF-1) in human Tau-A152T neurons. (**A**) Waterfall plot of effect of 10 µM compound of a subset of HMS LINCS kinase inhibitor library on p-Tau (PHF-1) in the cell body (left panel) or neuronal processes (right panel). Dashed (upper) line represent effect of DMSO (set at 1), dotted (lower) line represents effect of 10 µM CHIR-99021. Green dot represents no or minimal effect (≤ 20% decrease) of compound on neurite area, red dot represents adverse effect (> 20% decrease) of compound on neurite area. Values are mean ± SEM. (**B**) Chemical structure of CHIR-99021 and three of the hit compounds, Tozasertib, AT7519 and CGP-60474. (**C**) Concentration response curves of CHIR-99021 and three of the hit compounds on p-Tau (PHF-1). The compound hits, AT7519 and CGP60474, dose-dependently decreased the mean p-Tau (PHF-1, Tau-pS396/pS404) intensity in both the neuronal processes and cell body more potently than CHIR-99021 (our positive control in the assay), whereas Tozasertib only dose-dependently decreased the mean p-Tau intensity in neuronal processes. Each point is the mean ± SEM of four biological replicates.

Given the promising results described above testing candidate compounds, we implemented this high-content imaging platform assay using the patient Tau-A152T cell line to screen small molecule libraries for modulators of tau phosphorylation and levels. Beginning with a custom collection of 44 kinase inhibitors screened at a concentration of 10 µM, we observed varying effects on mean PHF-1 intensity in the cell body and neuronal processes (Fig. 8A). By setting a threshold for decreased PHF-1 mean intensity in both the cell body and neuronal processes to be greater than that observed with CHIR-99021 treatment, we identified two compounds, AT7519 and CGP-60474 (chemical structures in Fig. 8B) that were strongly active. Also, among the 44 kinase inhibitors tested, Tozasertib had a unique subcellular localization-dependent effect on p-Tau; that is, this compound decreased mean PHF-1 intensity in neuronal processes but had minimal effect on PHF-1 intensity in the cell body (Supplementary Fig. S10). The effects of AT7519, CGP-60474 and Tozasertib were confirmed when we tested these compounds at multiple concentrations using new stocks that we obtained from independent commercial sources (Fig. 8C). Taken together, these data demonstrate that our high-content human tau imaging assay can be applied to screening compound libraries for novel therapeutics for mutation- and patient-specific forms of tauopathies.

## Discussion

In developing a high-content image-based assay in a human neuronal cell model of tauopathy suitable for screening purposes, we needed to accomplish two objectives: (1) large-scale rapid generation of reproducible (low variability) differentiated neuronal cultures from healthy and patient-derived iPSC that express physiological or pathology-relevant endogenous levels of tau, respectively, and (2) develop an image analysis pipeline that can quantitatively measure tauopathy-relevant phenotypes in a morphologically complex neuronal culture system. We utilized a doxycycline-inducible Ngn2 vector incorporated into either iPSC or NPC to rapidly and efficiently generate excitatory glutamatergic forebrain neurons. These cells acquire neuronal gene expression patterns and morphology within 2 weeks and can become electrically responsive to glutamate receptor antagonists within 3 weeks^30^. We further simplified the handling of these cells by the stable integration of the iNgn2 vector into NPC (iNgn2-NPC), allowing for differentiation of relatively large quantities of neurons in microwell-plate format, and using a single type of cell medium (N3aM). Another method using the Ngn2 system to differentiate iPSC-derived wild-type cells for screening assays has been recently described^31^, but this system still requires a 2-step culturing protocol involving trypsinization and replating after the start of differentiation and then over of a month of additional neuronal differentiation before compound treatment.

In order to automate the quantification of fluorescence signals specifically in cell bodies and neuronal processes in Ngn2-differentiated neurons, we needed to develop an image analysis algorithm that could deal with the complexity and density of human neuronal cell cultures. Two features of our neuronal cultures that presented difficulties for the imaging analysis algorithm were clumping of neurons, and also instances in which neuronal processes crossed over cell bodies. Clumping of cells prevented accurate detection of individual cell parts, and thus our algorithm excludes these clumps. For the same reason, instances of neuronal processes crossing over cell bodies were excluded from analysis. These and other measures enabled us to detect fluorescence signals specifically in cell bodies and neuronal processes in our human Ngn2-differentiated cultures, which represents, to our knowledge, unprecedented levels of neuronal density for cultures used in high-content screening, in comparison to previous work on automated segmentation approaches for rodent-derived neuronal cultures (e.g.^52,53^). Additionally, with the goal of rapidly screening large collections of small molecules, our aim was to achieve image acquisition and segmentation in a single, microwell oriented platform, which was afforded by the IN Cell Analyzer 6000 imaging system. This imaging system collects data in an unbiased manner from the vast majority of the area in each well, allowing for a very high level of non-subjective neuron sampling and the resulting robust quantitation of fluorescence signals with high statistical power.

Here we demonstrate that our imaging assay can be used to screen for small molecules that reverse tau phenotypes associated with disease, evident in human tauopathy patient iPSC-derived neurons. Specifically, we used iPSC-derived neurons from a tauopathy patient with a *MAPT* missense mutation encoding A152T. We chose to study this tau variant because studies using transgenic animal models (zebrafish, mice) expressing Tau-A152T show that p-Tau and/or total tau are elevated^48,54–56^. These Tau-A152T phenotypes can also be observed in human iPSC-derived neurons. For example, in a previous study that utilized genetic editing of a heterozygous Tau-A152T iPSC line to create isogenic homozygous Tau-A152T and corrected into homozygous wild-type tau lines, there was an increase in the number of p-Tau positive iPSC-derived neurons from barely detectable levels in homozygous-corrected wild-type, some in heterozygous Tau-A152T, and the most in homozygous Tau-A152T cells. This gene-dose dependent effect was also seen with the degree of neurodegeneration of the iPSC-derived cells^57^. We have previously shown that iPSC-derived Tau-A152T neurons harbour a larger p-Tau and total tau load than control neurons^27^. Indeed, we have previously found Tau-A152T-associated increases in total and p-Tau in iPSC-derived neurons when comparing several Tau-A152T and control cell lines^27^, which includes the control and Tau-A152T cell lines utilized in the current study. Furthermore, these Tau-A152T neurons are more vulnerable to exogenous cellular stressors, and this increased sensitivity can be rescued by reducing tau protein levels, either by gene-editing or pharmacological activation of autophagy. These observations of increased tau load and enhanced sensitivity to stressors occur despite the fact that human iPSC-derived neurons such as the iNgn2 neurons described in this study predominantly express 3R tau, suggesting that these cultured human neurons have not attained the maturity of neurons in the healthy adult human brain which express in equal parts 3R and 4R tau. Thus, when comparing phosphorylation levels between iPSC-derived neurons and post-mortem human tissue it is also important to consider that tau phosphorylation is developmentally regulated^58^. The extent to which the biochemical mechanisms underlying the developmentally regulated changes in tau phosphorylation in human iPSC-derived neurons are the same as those perturbed in the context of neurodegeneration needs to be clarified in future studies with systematic comparisons across human fetal and adult autopsy brain tissue^59^. Therefore, although iPSC-derived neurons are immature compared to neurons in the human adult brain, they do exhibit pathological changes of tauopathy, and therefore constitute an important, pathophysiologically relevant cell model of neurodegeneration.

We have observed that iPSC-derived Tau-A152T neurons do not stain with Thioflavin-S, and thus do not contain tau aggregates. The Tau-A152T variant does not tend to form aggregates, but it does have a tendency to form oligomers, and it is the soluble or oligomeric form of Tau-A152T that is pathogenic^13,60^. Our Tau-A152T neurons, as well as numerous transgenic Tau-A152T animal models (zebrafish, mice^48,54–56^), show increased tau load (shown in western blots in Fig. 7 in this study and by mass spec^27^). As described above, this increased tau load makes the neurons more susceptible to external stressors and this sensitivity is abrogated by knockdown of tau^27^. We use the increased pathogenic load of tau in Tau-A152T neurons to discover potential therapeutic small molecules that can lower tau and p-Tau levels.

As a positive control for our assay, we used the GSK3 inhibitor CHIR-99021 to induce and test our ability to detect decreases in p-Tau since more than 40 putative phosphorylation sites in tau have been identified as targets of GSK3^61^. This use of CHIR-99021 as a GSK3-inhibiting positive control is further supported by our recent finding that a set of positron emission tomography (PET) ligands that potently inhibit GSK3β in vitro also reduce tau phosphorylation in neurons^51^. In addition to showing that CHIR-99021 reduced tau phosphorylation in our human glutamatergic neurons, we also observe that CHIR-99021 decreased tau levels (Fig. 7); these effects together may enhance the ability of this type of inhibitor to antagonize tau pathology. We also utilized a mass spectrometry-based kinome screen to determine the set of kinases inhibited by CHIR-99021 in our human NPC and neurons; this assay showed that CHIR-99021 was highly selective for GSK3α and GSK3β, especially in neurons. The reduction in tau and p-Tau levels produced by GSK3β inhibitors demonstrated here may also have relevance for pharmacological strategies to prevent tauopathy in early stages of disease. A key early stage pathological event may be the intercellular propagation of pathogenic tau in a prion-like protein transmission across the brain. It has been shown that it is the PBS-soluble, p-Tau from AD patients that is taken up by neurons and p-Tau antibodies, including one targeting tau-pS396, are effective in reducing tau uptake^8,62^. A number of kinases can phosphorylate the PHF-1 epitope (S396/pS404), including GSK3 and CDK5^63^. Both the protein levels and activity of GSK3 in the brain of tauopathy patients appear to correlate with the progression of neurodegeneration (reviewed in^61^). Two GSK3 inhibitors, tideglusib, and lithium, have gone through phase II clinical trials for AD and/or PSP but were halted due to poor tolerability and/or failure to meet the primary endpoints^64^. However, one concern about these previous clinical studies is whether the treatment was given early enough in the disease process to have a disease-modifying effect; that is, to treat at an early stage to prevent cell loss and brain atrophy. Recent developments in radioligands such as [^18^F]AV1451^65^ that can detect and track the progression of tau pathology in living patients will aid in diagnosing tauopathy patients at earlier stages of the disease.

Not only could a GSK3-selective inhibitor produce a decrease in tau phosphorylation in our assay, but in addition, the multi-kinase inhibitors enzastaurin and ruboxistaurin^49^ also produced decreases in p-Tau, suggesting that it may be possible to target multiple kinases to achieve a reduction in tau phosphorylation in human neurons. Since these inhibitors have been in clinical trials, including in the case for ruboxistaurin of peripheral diabetic neuropathy^66^, haematological cancers for enzastaurin^67^, this raises the possibility of repurposing them for tauopathy therapy. Lastly, we performed a pilot screen with a small library of kinase inhibitors and found AT7519 and CGP-60474 as two additional small molecules capable of reducing tau phosphorylation in human neurons to an extent that was greater than the positive control CHIR-99021. While both AT7519 and CGP-60474 are known as cyclin dependent kinase (CDK) inhibitors, they also bind to other kinases, predominantly within the CMGC family^49^ (KINOMEscan LINCS HMS website). Further elucidation of their precise mechanism of action and relevant targets has the potential to provide a lead for developing tauopathy therapeutics.

In this study, we showed that not only do the kinase inhibitors CHIR-99021 and enzastaurin decrease tau phosphorylation, but they also decrease the level of total tau, suggesting that these compounds can affect tau clearance in human neurons. Indeed, we have previously shown that iPSC-derived Tau-A152T neurons have an increased tau load and are more susceptible to external stressors, which can be attenuated pharmacologically with an autophagy inducer^27^. Tau can be degraded via either autophagy and/or ubiquitin-proteosome pathways^68,69^ (reviewed in^70^), but when Tau-A152T is overexpressed in either cell and animal models, there is disruption of these clearance pathways in clearing this mutated form of tau^26,56^. It was further shown that phospho-mimicking residues engineered into tau located either in the AT8 or PHF-1 epitopes severely reduced the binding of tau to lysosomes^26^, impacting its ability to be cleared from the cell. Taken together, these results suggest that kinase inhibitors could be beneficial in not only decreasing the phosphorylation of tau per se, but also aid in clearing aberrant tau from human neurons.

Although to date we developed this assay in cells from a tauopathy patient with one specific tau mutation, the procedure we describe here could be used to develop similar assays for other tau mutations in cells from patients with any of the various forms of tauopathies. Such assays could then be used to screen compound libraries for novel therapeutics for different tauopathies. This would be advantageous if compounds differ in effectiveness at reducing tau pathology arising from distinct tau mutations. As demonstrated in this study and elsewhere^31,71^, the strategy of screening compounds that are either FDA-approved or currently in pre-approval clinical trial investigation to find novel therapeutics for tauopathy is now highly feasible. For instance, it is exciting to find that enzastaurin and ruboxistaurin, two compounds in clinical trials, can decrease tau in our human neurons; this raises the possibility of rapidly repurposing these or related drugs for tauopathy therapy. In future work, we plan to screen the full set of FDA-approved drugs and other bioactive compounds in our high throughput tau/p-Tau imaging assay with the objective of creating new chemical tools for the tauopathy field and potential leads for novel therapeutic development.

## Methods

### Generation of iNgn2 lentiviruses

Using methods described previously^34,72^, the Tet-O-Ngn2, rtTA, and Tet-O-Ngn2-BSDR lentiviruses were produced in HEK293T cells by cotransfecting the pTet-O-mNgn2-puro plasmid (gift from the Wernig lab), the transactivator plasmid pFUW-M2rtTA (Addgene) or pTetO-mNgn2-puro-PGK-BSDR plasmid (made by inserting a blastocidin S deaminase resistance gene (BSDR) driven by the human PGK promoter into pTet-O-mNgn2-puro plasmid as described previously^34^) with the helper plasmids pCMV-dR8.2 dvpr (Addgene) and pMD2.G (Addgene) with Lipofectamine 2000 (ThermoFisher Scientific).

### Ngn2 transduction and induction in iPSC and NPC

The control iPSC lines, 8330-8 and CTR2-17, and the Tau-A152T iPSC line, 19-5 (generation of these iPSC lines was described previously^73–75^ and the NPC lines, 8330-8-RC1 and 19-5-RC6 (derived from 8330-8 and 19-5 iPSC line, respectively; derivation method previously described^34^) were used in this study. The two iPSC/NPC cell lines used in this study, the control line (8338-8/8330-8-RC1), and the Tau-A152T line (19-5/19-5RC6) were the same cell lines as used in Silva et al.^27^ (referred to as 8330-8-RC1 and 19-L5-RC6). NPC are capable of dividing at a stable rate up to 70 passages in standard culture conditions, and can be differentiated into neuronal and glial cell types. Approval for work with human subjects’ materials (iPSC and iPSC-derived cells) was obtained under IRB-approved protocols at Massachusetts General Hospital/Mass General Brigham (2009P002730) and University of California, San Francisco (10-00234). Both control (8330-8/8330-8-RC1) and Tau-A152T (19-5/19-5-RC6) cell lines were used in the development and characterization of the high-content imaging assay with similar results; for simplicity, only data for the control cell line is shown in Figs. 1, 2A–C, 3, 5, and Supplementary Figs. S1–S3, S6. To start the acute transduction and induction of iPSC or NPC with Ngn2, on day -1, the cells were plated and transduced with lentiviruses (see Fig. 1). iPSC were grown on Matrigel-coated plates and fed daily with mTeSR1 media (Stemcell Technologies). Healthy iPSC cultures, with little apparent differentiation were used for transduction and Ngn2 induction. Confluent healthy iPSC cultures were dissociated with Accutase and plated onto Matrigel-coated wells at a cell density of 2 × 10^5^ cells/cm^2^ (2 × 10^6^ cells into a well of 6-well plate) in mTeSR1 media containing 10 µM Y-27632 (EMD Millipore). NPC were grown in neural proliferation media (NPM, media components from Gibco unless otherwise noted: 70% DMEM, 30% Ham’s F12 (Mediatech), 2% B-27 neural supplement, 1% penicillin–streptomycin, 20 ng/mL bFGF (Stemgent), 20 ng/mL EGF (Sigma) and 5 µg/mL heparin (Sigma). Confluent NPC were dissociated with TrypLE and plated into wells coated with 20 µg/mL poly-ornithine and 5 μg/mL laminin (POL-coated, by sequential coating with poly-ornithine and laminin) at a cell density of 5 × 10^4^ cells/cm^2^ (5 × 10^5^ cells into a well of 6-well plate). The cells were transduced with the Tet-O-Ngn2 and rtTA concentrated lentiviruses 1 h after plating. Plates of transduced cells were centrifuged at 930×*g* for 30 min before returning the plates of cells to the incubator. Day 0 (24 h after the start of transduction) is the start of Ngn2 induction in which the media was changed to N3a neural iNgn2 media (N3aM^34^, 48% Neurobasal Medium, 48% DMEM/F12, 1% B27, 0.5% N2, 0.75% GlutaMax, 1% penicillin/streptomycin, 0.5% MEM NEAA, 50 µM β-mercaptoethanol (BioRad), 0.2% bovine serum albumin (Sigma), 2 µg/mL doxycycline (Clontech), 10 ng/mL BDNF (Peprotech) and 10 ng/mL NT3 (Peprotech)). To start Ngn2 induction of the iNgn2-NPC stable cells (generation of the iNgn2-NPC stable cells was previously described in detail^34^), at day 0 (see Fig. 1) the cells were seeded into plates coated with 20 µg/mL poly-ornithine and 5 µg/mL laminin (POLS-coated, simultaneous coating with poly-ornithine and laminin) containing N3aM. The iNgn2 cells were fed with fresh N3aM every two days, with the addition of 1 µg/mL puromycin at day 2 and 4 to select for cells expressing Ngn2-TA-puro, and the addition of 5 µM AraC (Sigma) at day 6 to inhibit any dividing cells (typically only a small percentage of cells in the cultures). Neurons in 96-well plates were fed with half replacement of fresh N3aM starting at day 2; neurons in 24-well and 6-well plates were refed with half replacement of fresh N3aM after day 6. For iNgn2 neurons fed with astrocyte-conditioned media (ACM), ACM was made by placing N3aM (minus bovine serum albumin, doxycycline, BDNF and NT3) over confluent human cerebellar astrocyte cultures (HCA, ScienCell Research Laboratories) for 24 h, and then collecting and centrifuging the ACM at 3700×*g* for 5 min to pellet cellular debris, and then storing aliquots of the supernatant at − 80 °C until use (ACM aliquots only undergo one freeze thaw cycle). The acutely transduced iNgn2 neurons were replated at day 4 by dissociating the cells with Accutase and replating on POLS-coated 12 mm glass coverslips (Chemglass Life Sciences) in 24-well plates at a cell density of 3 × 10^5^ cells/well or in 6-well plates at 8 × 10^5^ cells/well. Live cell phase contrast images at various iNgn2 time points were captured using a Zeiss Axiovert Observer.Z1 microscope and a 10× objective (NA 0.25) equipped with a Zeiss Axiocam MRm digital camera.

### Immunocytochemistry and image acquisition

Differentiated iNgn2 neurons were fixed with 4% formaldehyde (Tousimis) for 20–30 min at room temperature, washed once with PBS, and then incubated in blocking buffer (2% goat serum, 1% BSA, 0.1% gelatin, 0.1% Triton X-100 and 0.05% Tween-20 in PBS) for 1 h at RT. Primary antibody was added and cells were stained overnight at 4 °C at the following dilutions: 1:100 PHF-1 (pTau-S396/S404, generously provided by the late Peter Davies), 1:1000 K9JA (total Tau, Dako), 1:1000 βIII-tubulin (Synaptic Systems), 1:1000 Tau-pS396 (Invitrogen), 1:100 AT8 (pTau-pS202/T205, ThermoFisher Scientific), 1:1000 SMI312 (Covance), 1:2500 MAP2 (EnCor Biotechnology), 1:1000 VGLUT1 (Synaptic Systems), 1:2000 Synapsin 1 (Synaptic Systems) and 1:500 Homer 1 (Synaptic Systems). After the incubation with primary antibodies, the cells were washed with PBS and then incubated with the appropriate fluorophore-conjugated secondary antibodies and Hoechst 33342 (1:2500 dilution; Molecular Probes) in blocking buffer for 2 h at RT protected from light. Cells in 96-well plates were then washed and stored in PBS at 4 °C until imaging. Cells on glass coverslips were washed with PBS, mounted onto glass slides using ProLong antifade reagent (Molecular Probes) and stored protected from light until the mounting reagent had dried before imaging. Automated image acquisition was performed using the IN Cell Analyzer 6000 Cell Imaging System (GE Healthcare Life Sciences), a laser-based confocal high-throughput imaging platform with a Nikon Plan Fluor, ELWD (extra-long working distance), 20 × objective, 0.45 numerical aperture, Correction Collar 0–2.0, CFI/60. Images were captured with open aperture. To minimize photobleaching within a well, the 25 fields were spaced out beyond the visual field of the objective.

### High-content image analysis

Automated image acquisition of iNgn2 neurons in 96-well plates immunostained with PHF-1/K9JA/βIII-tubulin/Hoechst 33342 was performed by acquiring 25 fields evenly spaced over each well and at identical positions across all wells. Segmentation and quantification of the resulting images was performed using the Developer Toolbox of the IN Cell Analyzer 6000 system. Raw measures were derived from neuronal cell bodies and the neurite fields independently. A combination of Hoechst nuclear stain intensity and shape criteria, along with contiguous βIII-tubulin and K9JA signals were used to classify appropriately shaped objects as neuronal cell bodies. Neurites were identified and segmented using a combination of βIII-tubulin, K9JA, and PHF-1 signals. Once the cell body mask and the total cell mask (all neurites and all cell bodies) had been obtained, we employed ‘image math subtraction’ (similar to the correspondingly named ImageJ process) to remove cell body areas from the final neurite masks for quantification. Segmentation of images was performed within the proprietary Developer Toolbox software of the IN Cell Analyzer 6000. Given the inherent complexity of the neuronal morphology in our images, we found that an edge detection-based method worked better than intensity thresholding to create the initial cell body and neurite masks. Final criteria for inclusion/exclusion of objects were based on mean intensity of the entire object (for example the nuclei, which had substantially higher mean intensity in clumped, dead cells) rather than overall thresholding. Clumped, dead nuclei were identified and excluded from analysis during the segmentation process by a combination of size (dead nuclei are significantly smaller), Hoechst intensity (dead nuclei are significantly brighter), and morphological (dead nuclei usually have rugged shape rather than being oval or round with smooth circumference) parameters. We utilized 10 iterations to create the total neurite mask before fine tuning to remove cell body areas, clumped areas, and background. Images had to pass a number of quality control checkpoints: (a) Experimental 96-well plates were considered ‘unresponsive’ and thus excluded if PHF-1 raw intensity ratio for DMSO/CHIR-99021 (11 µM) < 1.3 based on extensive data collection over 3 years. (b) Individual images in each well with a neurite area smaller than the lower 95% Confidence Interval (CI) were excluded; (c) images with a neurite area smaller than the lower 95% Confidence Interval of the DMSO-treated wells were excluded. Mean PHF-1 intensity was calculated by taking the total PHF-1 intensity in an image field divided by the total neurite area of the same image. The mean PHF-1 intensity resulting from a compound treatment (fold change versus DMSO) was calculated by taking the mean PHF-1 intensity value in an image field divided by the average of the mean PHF-1 intensity of DMSO treatment of the entire well. PHF-1/K9JA ratio was calculated by dividing the mean PHF-1 intensity over the mean K9JA intensity for each image field. For enzastaurin and ruboxistaurin compound treatments, each dose of each compound was added to duplicate wells, and four DMSO wells were included in each plate. For the compound library and compound hits treatments, each dose of each compound was added to a single well per plate, with two replicate 96-well plates per culture. Dose–response data from the compound hits treatment were analysed by non-linear regression using GraphPad Prism and curve fitting was performed by plotting log (agonist) vs. response (variable slope) to determine the IC_50_ values of the compounds. Z-factors were calculated using Eq. (4) in Zhang et al.^76^ with 19 negative control (DMSO or vehicle-treated) wells and 20 positive control (11 μM CHIR-99021) wells for the control iNgn2 neurons and 20 wells for both negative and positive control wells for the Tau-A152T neurons.

### Small-molecule probes

CHIR-99021 (synthesized at Broad Institute), Enzastaurin (Selleck Chemicals), Ruboxistaurin (Sigma), AT7519 (Selleck Chemicals), Tozasertib (Selleck Chemicals) and CGP-60474 (Tocris). The HMS LINCS kinase inhibitor library was supplied to us as 2 µL 10 mM compound/well custom-arrayed in NUNC 96-well plate by the ICCB-Longwood Screening Facility.

### Western blotting

On the appropriate day, the cells used for western blots were washed once with PBS, detached from the wells using a cell lifter, and collected into a microfuge tube and centrifuged at 1300×*g* for 5 min before the cell pellet was frozen on dry ice before transferring to − 80 °C for storage. Cell lysates for immunoblotting were prepared one of two ways. One method was to resuspend the cell pellet in RIPA buffer containing a protease inhibitor cocktail (Complete Protease Inhibitors, Roche) and a phosphatase inhibitor cocktail (PhosSTOP, Roche). Protein amount estimation in these RIPA lysates was performed using the BCA protein assay (Pierce) and a SpectroMax Plus 384 microplate reader (Molecular Devices) driven by the Softmax Pro software. Lysate volumes containing equal amounts of total protein were added to sample buffer (NEB), incubated at 95 °C for 10 min before loading onto gels. The other method was to resuspend the cell pellet in approximately 3 × volume of hot sample buffer, incubate at 95 °C for 10 min with occasional mixing, and then loading equal volumes of sample onto gels. Unless otherwise noted, Nupage Novex 7% Tris–acetate pre-cast polyacrylamide gels (ThermoFisher Scientific) were used and the gels were run at 150 V for 1 h, and then the proteins were transferred to PVDF membrane (Fisher Scientific) using the XCell II Blot Module (ThermoFisher Scientific). Blots were rinsed in Tris-buffered saline with Tween (TBS-T), and then blocked with 5% BSA in TBS-T (blocking buffer) for 1 h at RT before incubating the blots with primary antibody in blocking buffer overnight at 4 °C, except for GAPDH which was incubated at RT for 1 h. The primary antibody used were at the following dilutions: 1:1000 TAU5 (Invitrogen), 1:1000 Tau-pS396 (Invitrogen), 1:2000 Synapsin 1 (Synaptic Systems), 1:1000 PSD-95 (clone 108E10, Synaptic Systems), 1:1000 GluR1 (Millipore), and 1:5000 GAPDH (Abcam). After the incubation with primary antibodies, blots were washed with TBS-T, and then incubated for 2.5 h in the appropriate horseradish peroxidase-coupled secondary antibody (Cell Signaling Technologies) at 1:2000 dilution. After washing the blot with TBS-T, the blot was incubated with ECL Western Blotting Substrate (Pierce) for 1 min before it was blotted dry and exposed to autoradiographic film (Labscientific Inc). Bands were analysed using ImageJ (NIH). Uncropped blots with molecular weight reference are shown in Supplementary Figs. S11 and S12.

### RNAseq and analysis

On the appropriate day, media was removed from the cells, and then TRIzol reagent (ThermoFisher Scientific) was added directly to the cell culture plates. Cells were lysed in TRIzol for 5 min, and then the lysates were collected in microfuge tubes and stored at − 80 °C. RNA was isolated using the Direct-zol RNA miniprep kit (Zymo Research). rRNA subtracted and strand specific cDNA libraries were generated using the KAPA kit (KAPA Biosystems). A NextSeq 500 v2 (2 × 76 PE kit, Illumina) was used to sequence the RNA fragments at a read depth of > 87 × 10^6^ reads mapped per sample. Gene expression values were estimated by Kallisto, and transcript per million (TPM) values were aggregated for each gene. For each time point, the mean was derived from combining the gene expression values for each of the control cell lines, 8330-8 and CTR2-17 (n = 2 biological replicates).

### Multiplexed inhibitor bead (MIB) chromatography, mass spectrometry (MS) and analysis

8330-8-RC1 proliferative NPC were plated at 4 × 10^5^ cells/well into POL-coated 6-well plates and starting the next day treated with 1 μM CHIR-99021 or DMSO for 24 h. For generation of iNgn2 neurons, 8330–8-RC1 iNgn2-NPC stable cells were plated at 8 × 10^5^ cells/well into POLS-coated 6-well plates, and iNgn2 induction and cell maintenance was performed as described above. 13–14 day iNgn2 neurons were treated with CHIR-99021 (1 μM) or DMSO for 24 h before harvesting the cells. The cells were washed twice with cold PBS, and then harvested on ice by addition of ice-cold MIB lysis buffer (containing 50 mM HEPES, 150 mM NaCl, 0.5% Triton X-100, 1 mM EDTA, 1 mM EGTA at pH 7.5, 10 mM NaF, 2.5 mM Na_3_VO_4_, 1× cOmplete Protease Inhibitor Cocktail (Roche), 1 × Phosphatase Inhibitor Cocktail 2 (Sigma) and 1 × Phosphatase Inhibitor Cocktail 3 (Sigma)). The cell lysate was collected into 15 mL conical tubes and flash frozen in liquid nitrogen and stored at − 80 °C until MIB-MS analysis. The MIB-MS methodology has been described previously^45–47^. Specifically, lysates were clarified by centrifugation, and syringe-filtered (0.22 µm) prior to Bradford assay quantitation of concentration. Equal amounts of total protein were gravity-flowed over multiplexed inhibitor bead (MIB) columns in high salt MIB lysis (1 M NaCl). The MIB columns consisted of 175 µL mixture of six Type I kinase inhibitors (CTx0294885, VI-16832, PP58, Purvalanol B, UNC-21474, and UNC-8088A) custom-synthesized with hydrocarbon linkers and covalently linked to ECH-Sepharose (or EAH-Sepharose for Purvalanol B) beads as previously described^46^. Columns were washed with 5 mL of high salt (1 M NaCl), 5 mL of low salt (150 mM NaCl) MIB lysis buffer, and 0.5 mL low-salt lysis buffer with 0.1% SDS. Bound protein was eluted twice with 0.5% SDS, 1% beta-mercaptoethanol, 100 mM Tris–HCl, pH 6.8 for 15 min at 100 °C. Eluate was treated with DTT (5 mM) for 25 min at 60 °C and 20 mM iodoacetamide for 30 min in the dark. Following spin concentration using Amicon Ultra-4 (10 k cut-off) to ~ 100 µL, samples were precipitated by methanol/chloroform, dried in a speedvac and resuspended in 50 mM HEPES (pH 8.0). Tryptic digests were performed overnight at 37 °C, extracted four times with 1 mL ethyl acetate to remove detergent, dried in a speed-vac, and peptides further cleaned using C-18 spin columns according to manufacturer’s protocol (Pierce). Peptides were resuspended in 2% ACN and 0.1% formic acid. 40% of the final peptide suspension was injected onto a Thermo Easy-Spray 75 μm × 25 cm C-18 column and separated on a 180 min gradient (5–40% ACN) using an Easy nLC-1000. The Thermo Q Exactive mass spectrometry ESI parameters were as follows: 3e6 AGC MS1, 80 ms MS1 max inject time, 1e5 AGC MS2, 100 ms MS2 max inject time, 20 loop count, 1.8 m/z isolation window, 45 s dynamic exclusion. Raw files were processed for label-free quantification by MaxQuant LFQ^77^ using the Uniprot/Swiss-Prot human database and default parameters were used with the following exceptions–only unique peptides were used, matching between runs was utilized with fixed modifications: carbidomethyl (C) and dynamic modifications: acetyl (K), acetyl (Protein N-term), deamidation (N), oxidation (M), and phospho (STY) included. LFQ intensities were log2-transformed and used for comparison and peptide intensities for GSK3A and GSK3B were also plotted.

### GSK3 kinase assay

The details for the ADP-Glo kinase assay for GSK3β have been described previously^78,79^. The data were analysed by non-linear regression using GraphPad Prism and curve fitting was performed by plotting log (inhibitor) vs. response to determine the IC_50_ values of the compounds.

### Statistical analysis

Statistical analyses were performed using GraphPad Prism software. Unpaired t-tests were performed when comparing two groups of data, and p values are shown in the figure legends. One-way ANOVA was performed when comparing more than 2 groups of data, and the Dunnett or Šídák correction was used for multiple post-hoc comparisons; p values are shown in the figure legends.

## Supplementary Information

Supplementary Information.

## Data Availability

The datasets generated during and/or analysed during the current study are available from the corresponding authors on reasonable request.
