# Supplementary material for: High-content image-based analysis and proteomic profiling identifies Tau phosphorylation inhibitors in a human iPSC-derived glutamatergic neuronal model of tauopathy
Source: Sci Rep. 2021 Aug 23;11:17029. doi: 10.1038/s41598-021-96227-5 (PMC8382845; doi:10.1038/s41598-021-96227-5)
Supplement: Supplementary file 1 — Supplementary Information. [file 41598_2021_96227_MOESM1_ESM.pdf]

High-Content Image-Based Analysis and Proteomic Profiling Identifies Tau Phosphorylation Inhibitors in a Human iPSC-Derived Glutamatergic Neuronal Model of Tauopathy

Chialin Cheng[1]*, Surya A. Reis[1], Emily T. Adams[1], Daniel M. Fass[1], Steven P. Angus[2,11], Timothy J. Stuhlmiller[2], Jared Richardson[3], Hailey Olafson[4], Eric T. Wang[4], Debasis Patnaik[1], Roberta L. Beauchamp[5], Danielle A. Feldman[6], M. Catarina Silva[1], Mriganka Sur[6], Gary L. Johnson[2], Vijaya Ramesh[5], Bruce L. Miller[7], Sally Temple[8], Kenneth S. Kosik[9], Bradford C. Dickerson[10], & Stephen J. Haggarty[1,10]*

Supplementary Information

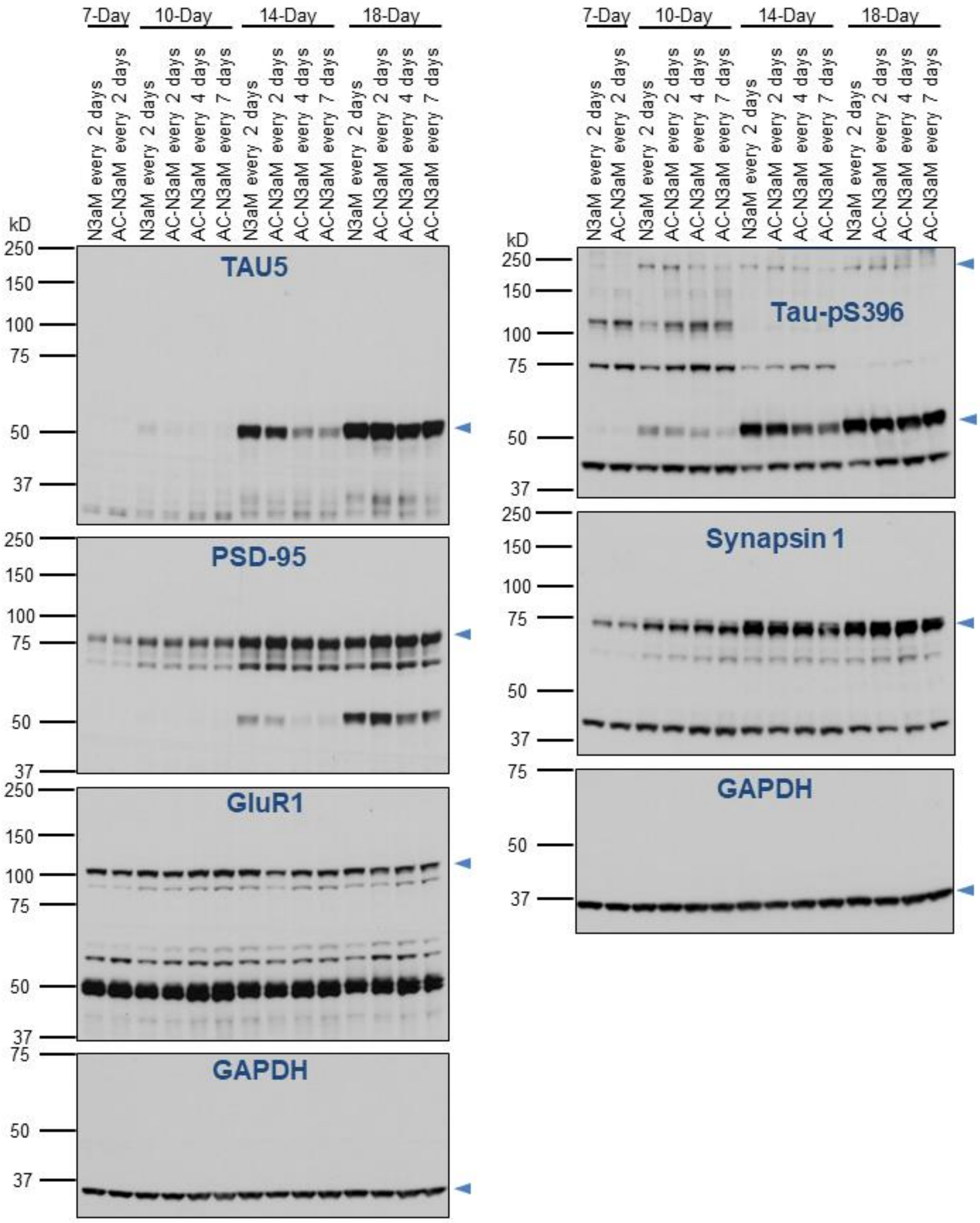

Supplementary Fig. S1. Optimization of cell feeding schedule. Examining the protein levels of total tau (TAU5), p-Tau (Tau-pS396), synaptic proteins PSD-95 and Synapsin 1 and a glutamate receptor (GluR1) in iNgn2 neurons at various time points after feeding them with fresh N3aM every 2, 4 or 7 days with or without prior astrocyte-conditioning (AC) of the media.

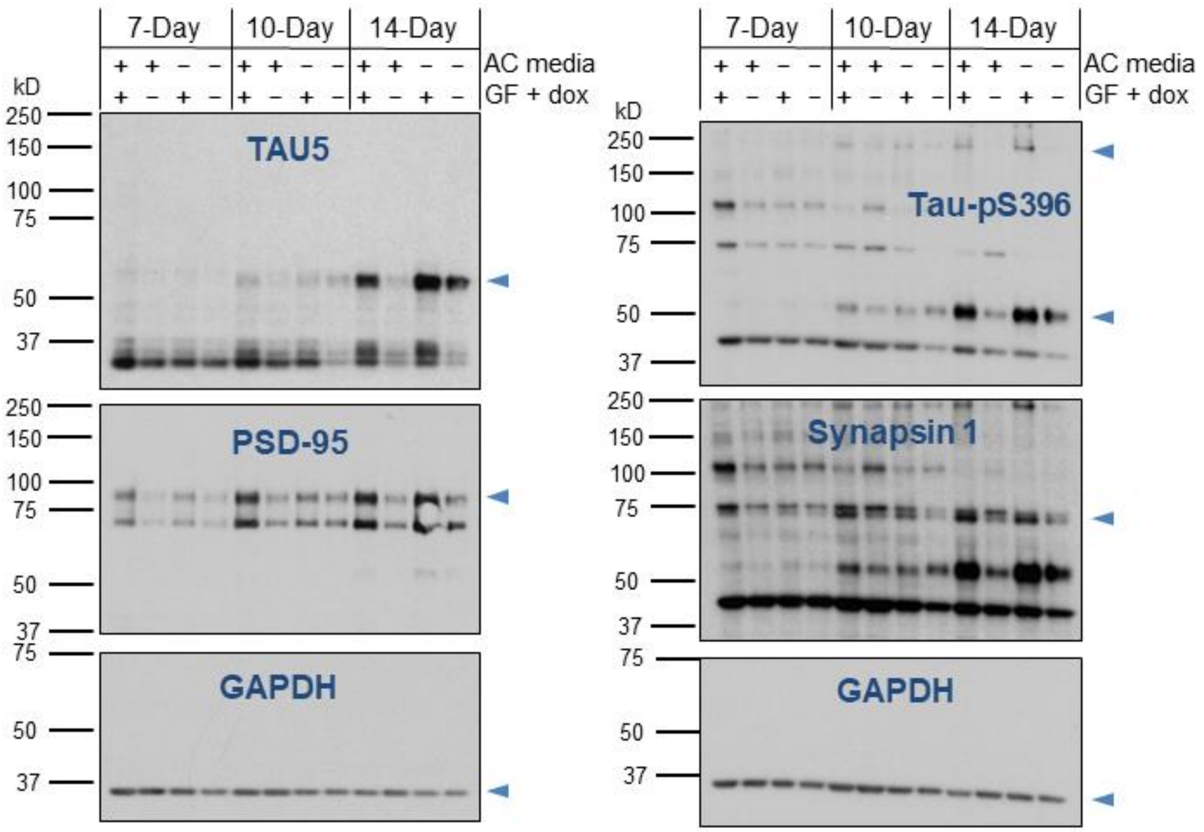

Supplementary Fig. S2. Effect of media additives on tau and synaptic protein levels. Examining the effect of astrocyte-conditioned media (AC media), doxycycline and the growth factors BDNF and NT3 (GF + dox) on protein levels of total tau (TAU5), p-Tau (Tau-pS396) and synaptic proteins PSD-95 and Synapsin 1 in iNgn2 neurons at various time points by western blot.

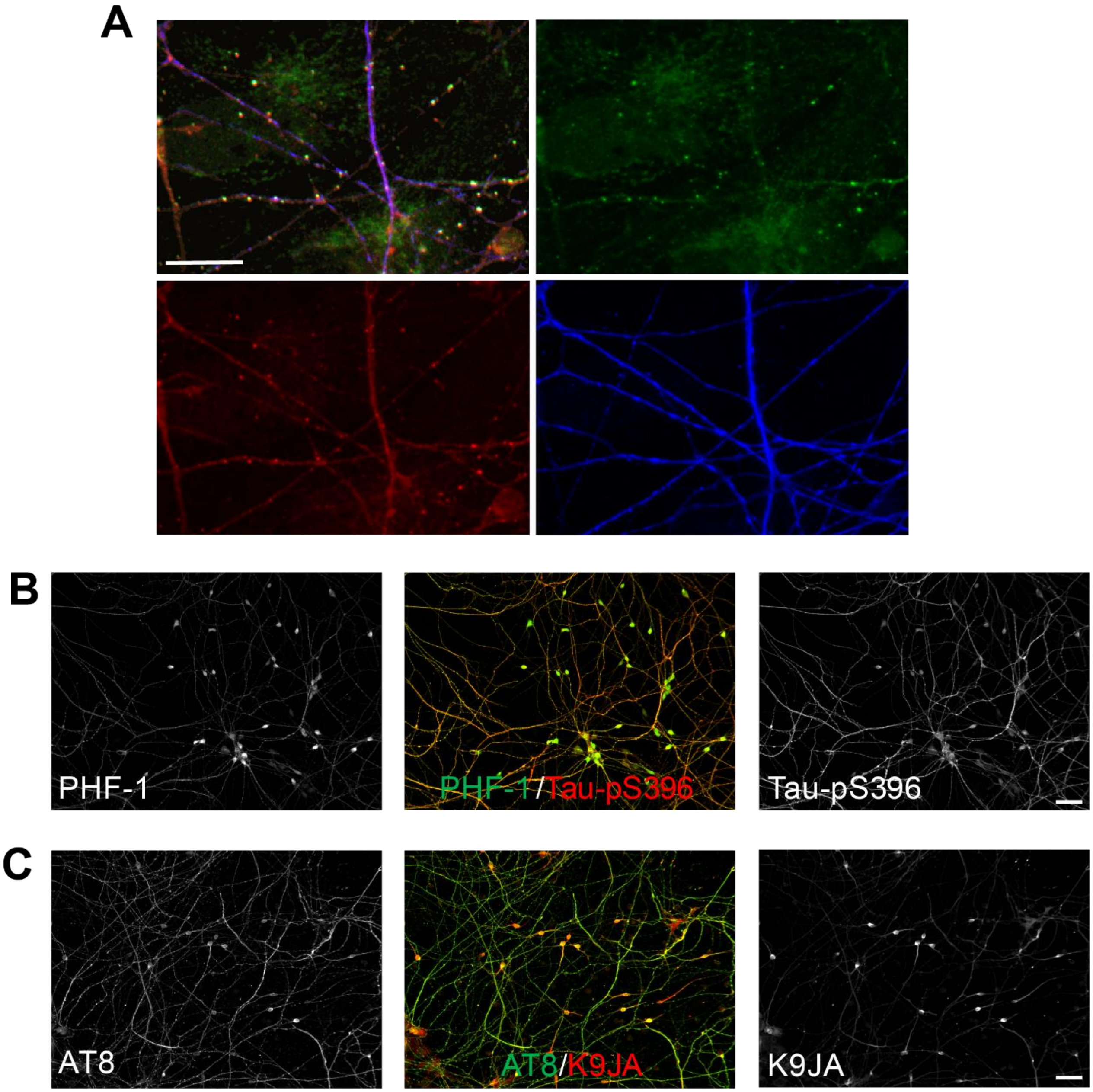

Supplementary Fig. S3. Immunocytochemical staining patterns of synaptic and tau antibodies in iNgn2 neurons. (**A**) Colocalization of the synaptic proteins VGLUT1 (green) and Synapsin 1 (red) along neuronal processes immunostained with the p-Tau antibody PHF-1 (blue). Scale bar represents 20 µm. (**B**) Comparison of cell staining pattern of two antibodies, PHF-1 and Tau-pS396, to the phosphorylated S396 residue in tau. (**C**) Cell staining pattern of another p-Tau antibody, AT8 (detects phosphorylation of tau at S202/T205). Scale bar represents 50 µm.

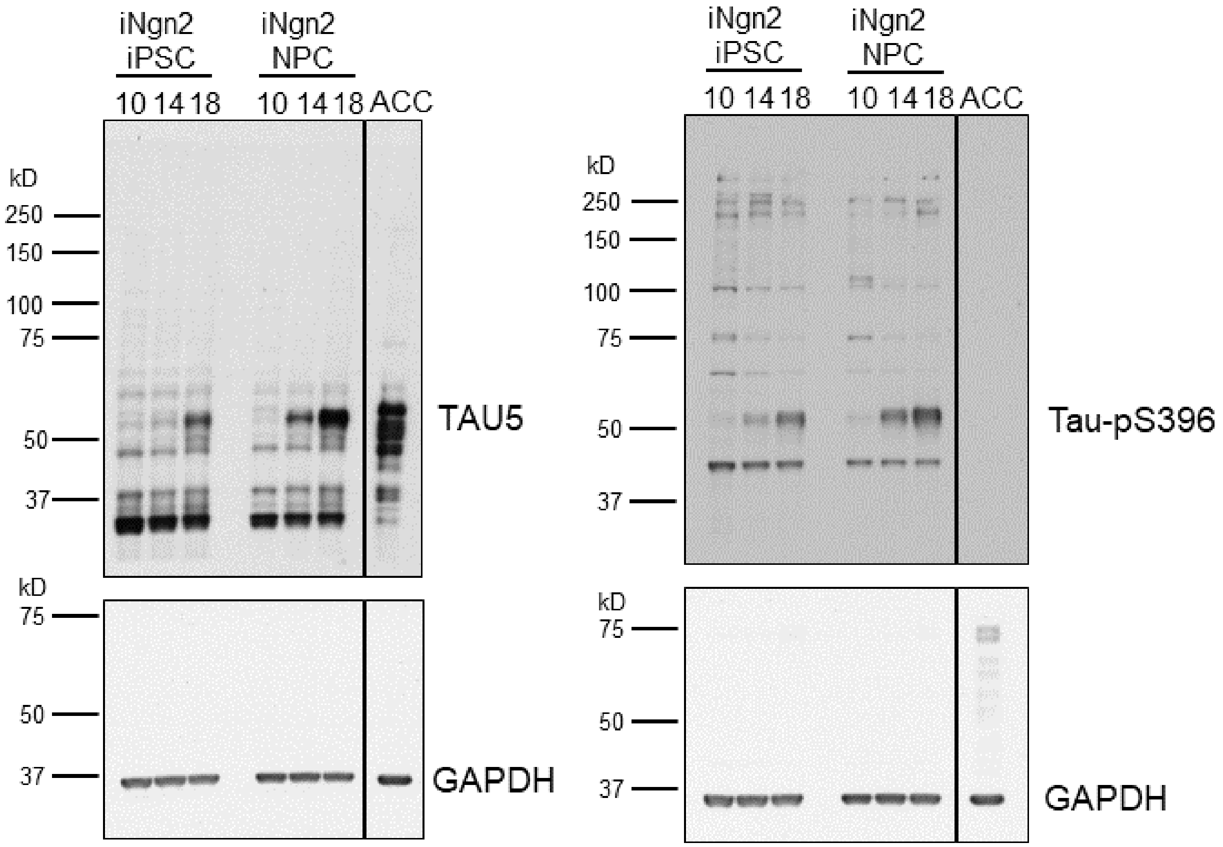

Supplementary Fig. S4. Expression of tau protein in Tau-A152T iNgn2 neurons as assessed by western blotting. Similar to control iNgn2 neurons in Fig. 3, there is increasing expression of total tau (TAU5) and p-Tau (Tau-pS396) throughout neuronal differentiation from transduction of iPSC or NPC, with robust levels in 14-day iNgn2 neurons. For comparison, human brain tissue from the anterior cingulate cortex (ACC) was also probed for tau expression. p-Tau was not detected in the ACC sample. iNgn2 neuronal samples and the ACC sample were run on the same blot, and blot lanes were cropped as indicated for clarity in the figure.

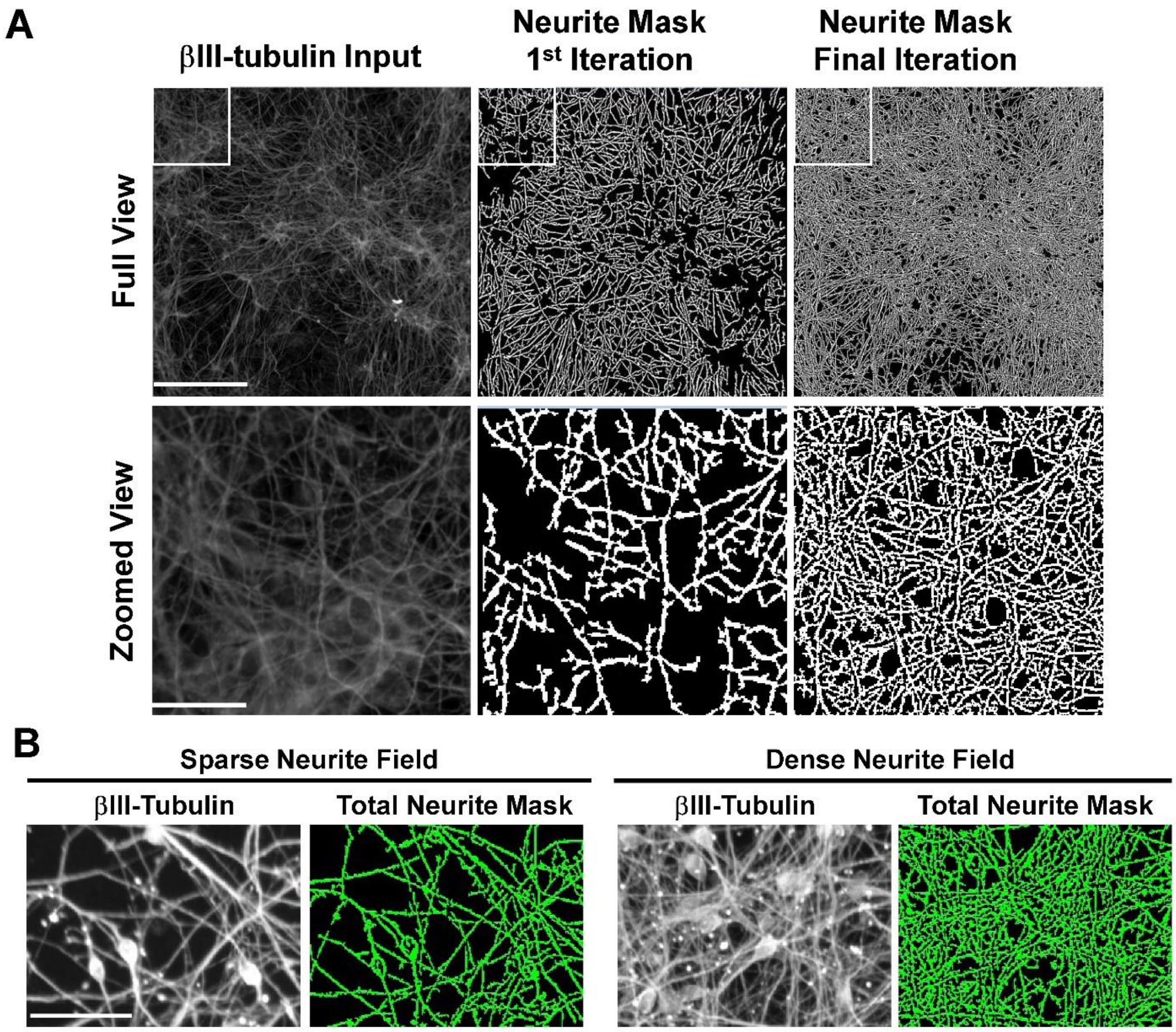

Supplementary Fig. S5. Image segmentation pipeline capabilities. **(A)** Comparison of 1st vs final iteration of neurite mask extraction during the multi-part, multi-iteration Image Segmentation Pipeline in a highly dense neurite field emphasizing the capability of the pipeline to deal with intra-image variability of neurite density, intensity, and background signal. Top: full image view. Bottom: Zoomed view. Scale bar in top panel represents 200 µm and 50 µm in bottom panel. (**B**) Demonstration of pipeline's ability to successfully segment both sparse (left) and dense (right) neurite fields in different images within the same analysis run. Scale bar represents 100 µm.

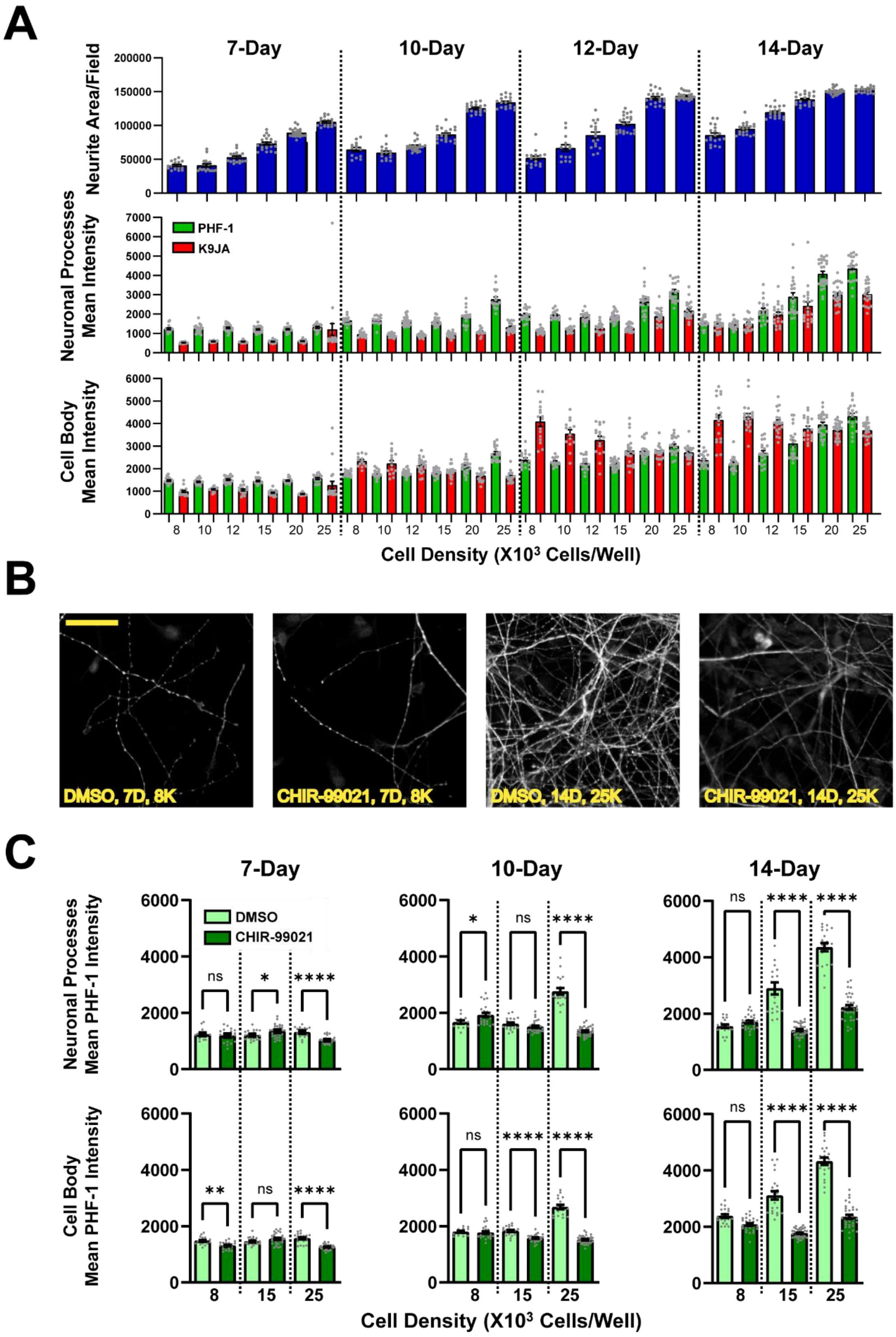

Supplementary Fig. S6. Figure legend continued on next page.

Supplementary Fig. S6. Optimization of assay parameters. (**A**) Neurite area, PHF-1 (p-Tau) and K9JA (total tau) mean intensity in cell bodies and neuronal processes of iNgn2 neurons at various time points after plating at the initial cell densities noted on the graph. Each bar represents mean±SEM; dots represent individual values. Density had a statistically significant effect on neurite area on all differentiation days (one-way ANOVA $p < 0.0001$). Density had a statistically significant effect on PHF-1 staining in neuronal processes and cell bodies on all differentiation days (one-way ANOVA $p < 0.05$ for cell body PHF-1 mean intensity on day 7; $p < 0.0001$ on all other days for both cell body and neuronal process mean intensity). Density also had a statistically significant effect on K9JA staining in neuronal processes and cell bodies on all differentiation days (one-way ANOVA $p < 0.01$ for neuronal processes and cell body K9JA mean intensity on differentiation day 7; $p < 0.01$ for cell body K9JA mean intensity on differentiation day 14; $p < 0.0001$ for both neuronal process and cell body K9JA mean intensity on all other differentiation days. (**B**) Representative images of p-Tau (PHF-1) immunostaining of 7-day (7D) and 14-day (14D) iNgn2 neurons initially plated at 8,000 cells/well (8K) or 25,000 cells/well (25K) treated with DMSO or 10 µM CHIR-99021. Scale bar represents 50 µm. (**C**) Effect of CHIR-99021 on mean PHF-1 intensity in cell bodies and neuronal processes of iNgn2 neurons at various time points after plating at the initial cell densities. Each bar represents mean±SEM; dots represent individual values. Statistical significance of the effect of CHIR-99021 on mean PHF1 staining intensity in neuronal processes and cell bodies at each plating density on each differentiation day was tested by one-way ANOVA followed by the Šídák post-hoc comparison testing and is indicated as follows: ns = not significant; * = $p < 0.05$; ** = $p < 0.01$; *** = $p < 0.001$; **** = $p < 0.0001$.

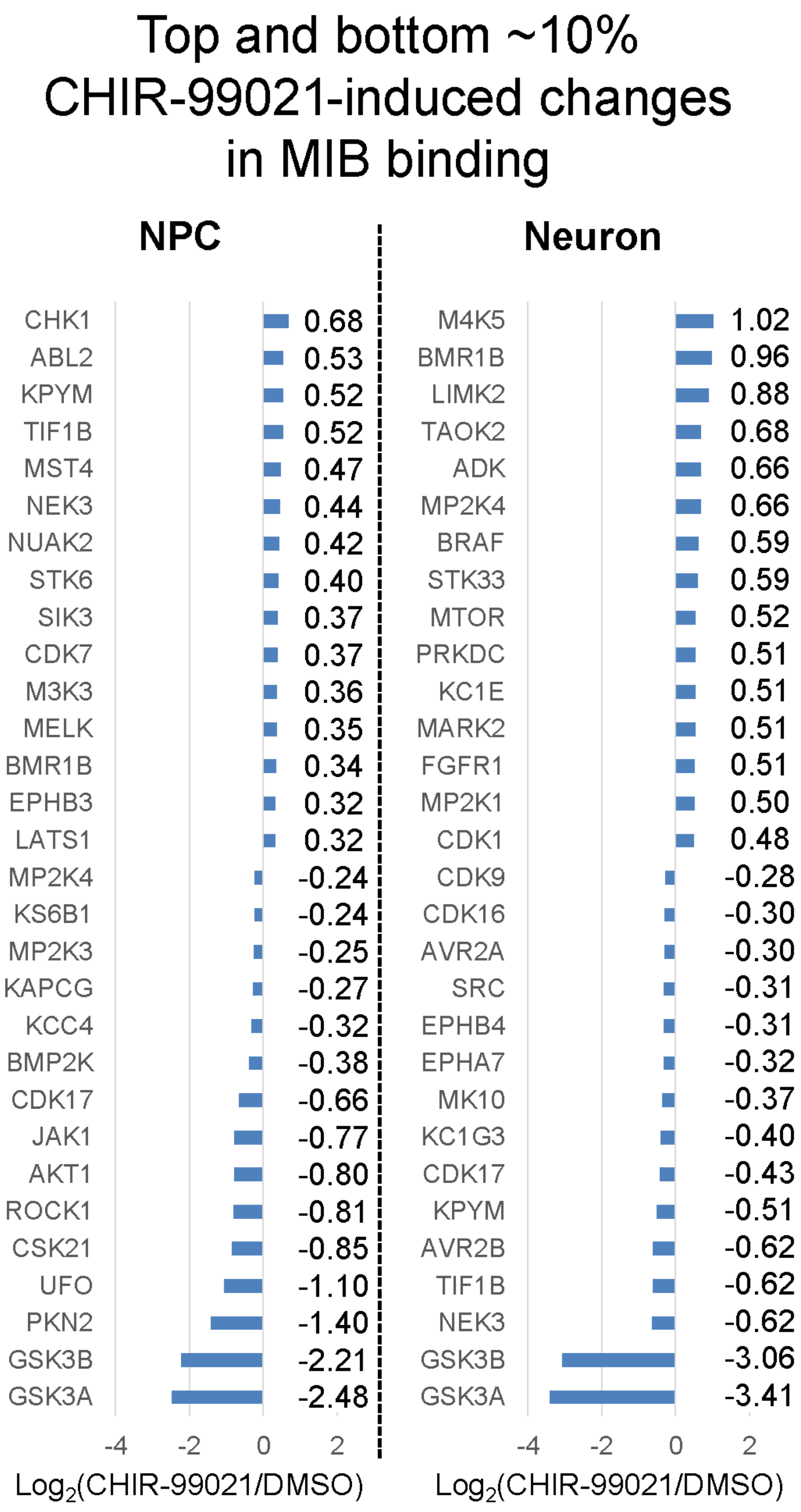

Supplementary Fig. S7. List of top and bottom 10% CHIR-99021-induced changes in the kinome of human NPC and neurons. The LFQ intensity values (normalized across all the mass spec runs and requires a minimum of 2 unique peptides) are expressed as $\log_2$ of CHIR-99021 induced fold change compared to DMSO.

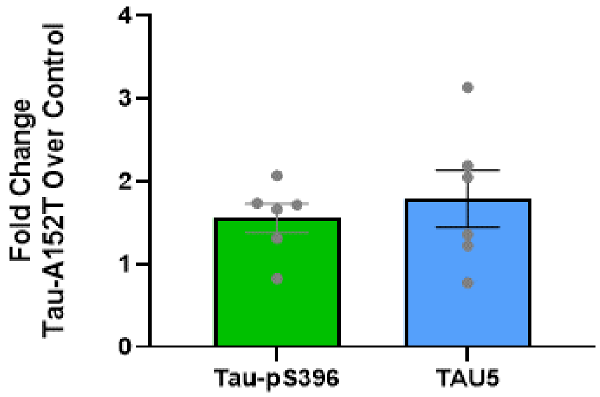

Supplementary Fig S8. Expression of p-Tau and total tau protein in control and Tau-A152T iNgn2 neurons. Quantification of p-Tau (Tau-pS396) and total tau (TAU5) immunoblots (with control and Tau-A152T samples in same blot) illustrates that p-Tau and total tau are increased in Tau-A152T neurons. Each bar represents mean±SEM; dots represent individual values.

Supplementary Table S1. Comparison of Mean Intensity in Neurite Versus Cell Body of iNgn2 Neurons

**Culture Batch**	**Ratio Neurite/Cell Body (Mean PHF-1 Intensity)**		**Ratio Neurite/Cell Body (Mean K9JA Intensity)**		**Neurite Area**	
	8330-8-RC1	***19-5-RC6***	***8330-8-RC1***	***19-5-RC6***	***8330-8-RC1***	***19-5-RC6***
**2015**	1.008±0.018	0.945±0.015	0.815±0.026	0.762±0.024	152565±895	156886±610 ***
**2016**	0.819±0.009	0.711±0.008 ****	0.403±0.007	0.335±0.007 ****	95361±991	101688±892 ****
**2017**	1.1±0.007	0.91±0.008 ****	0.838±0.011	0.587±0.011 ****	133525±495	133194±351

Notes:
Values are mean±SEM, unpaired t-test comparing 19-5-RC6 to respective 8330-8-RC1 (performed in Prism)
For 2015, one plate containing both cell lines (8330-8-RC1 n=20, 19-5-RC6 n=22)
For 2016, one plate of each cell line (8330-8-RC1 n=39, 19-5-RC6 n=44)
For 2017, four plates of each cell line (8330-8-RC1 n=233, 19-5-RC6 n=231)

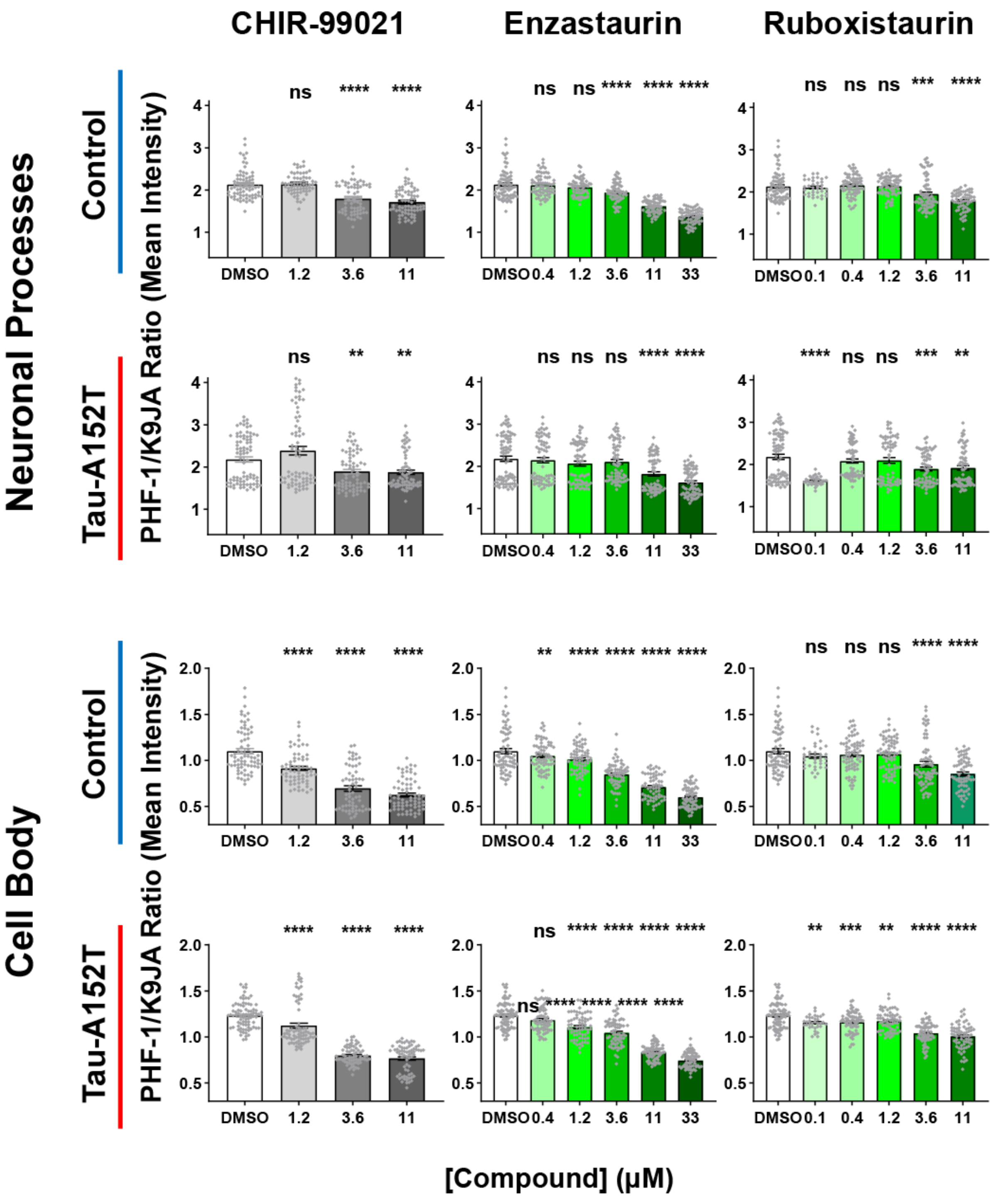

Supplementary Fig. S9. Effect of compounds on p-Tau/total tau ratio in human neurons. 20 hr treatment with CHIR-99021, enzastaurin and ruboxistaurin dose-dependently decreased PHF-1/K9JA mean intensity ratio in both cell bodies and neuronal processes in both control and patient iNgn2 neurons. Mean±SEM of two biological replicates; dots represent individual values. One-way ANOVA, Dunnett's post hoc test, ns=not significant, *p≤0.05, **p≤0.01, ***p≤0.001, ****p≤ 0.0001 compared to DMSO.

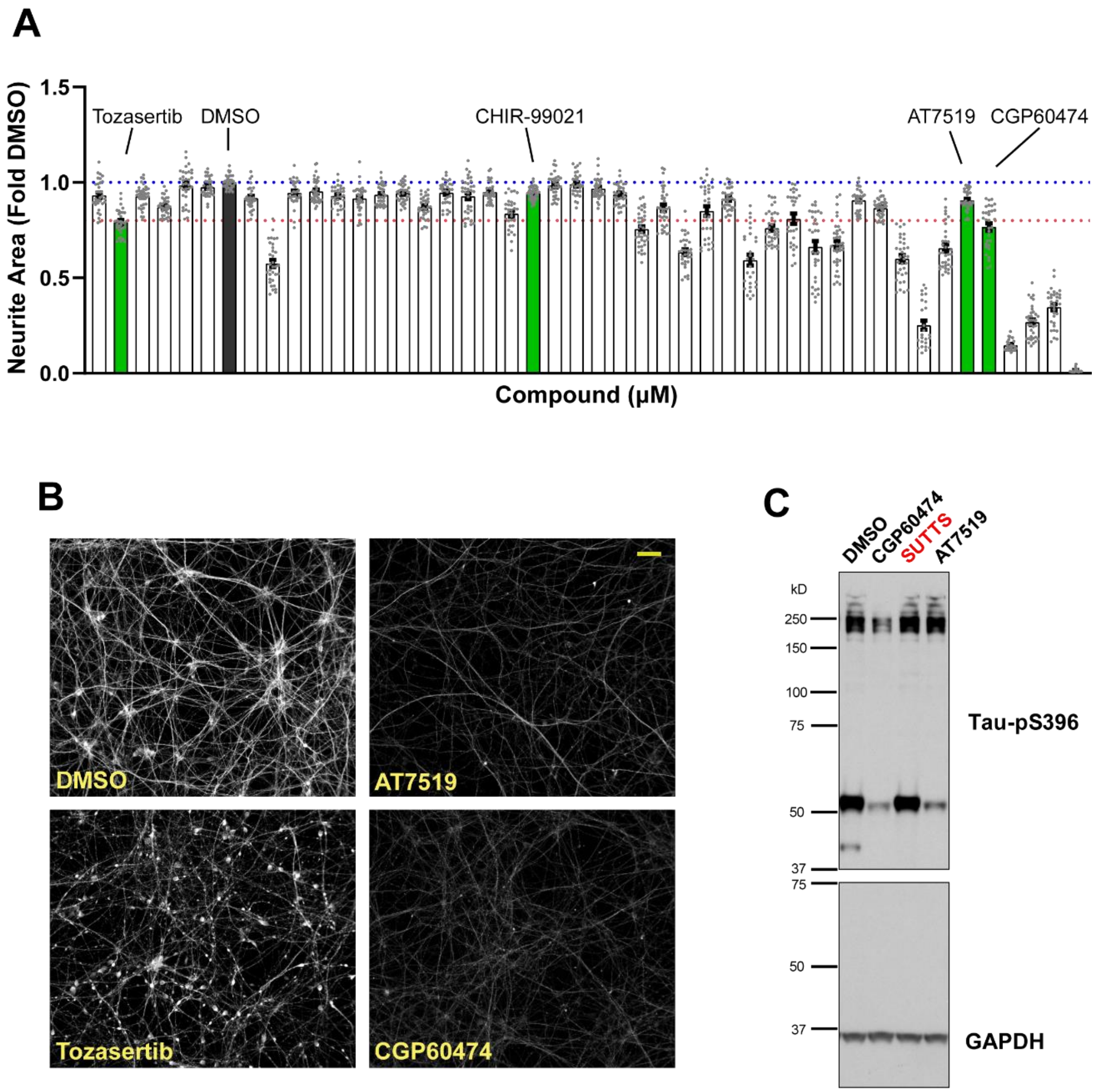

Supplementary Fig. S10. Additional data on the effect of compounds from a custom collection of 44 small molecule kinase inhibitors on p-Tau (PHF-1) in human Tau-A152T neurons. (**A**) Effect of 20 hr treatment of 10 μM compound on the neurite area of iNgn2 neurons (compound ordering in graph similar to order in left panel of Fig. 8A), with DMSO highlighted as the black bar, and compound hits described in Fig. 8 highlighted in green. Each bar represents mean±SEM; dots represent individual values. Compounds categorized as having no or minimal effect on neurite area are denoted between the blue and red dotted lines. (**B**) Representative images of PHF-1 (p-Tau) immunostaining in Tau-A152T neurons treated for 20 hr with DMSO or the hit compounds. There is a decrease in p-Tau in both the cell body and neuronal processes with AT7519 and CGP60474, whereas Tozasertib only decreases p-Tau in the neuronal processes. Scale bar represents 50 μm. (**C**) Representative western blot of the effect of the kinase inhibitors AT7519 and CGP60474 in decreasing p-Tau (Tau-pS396). The blot contains an uncropped lane with sample unrelated to this study (SUTTS) between CGP60474 and AT7519.

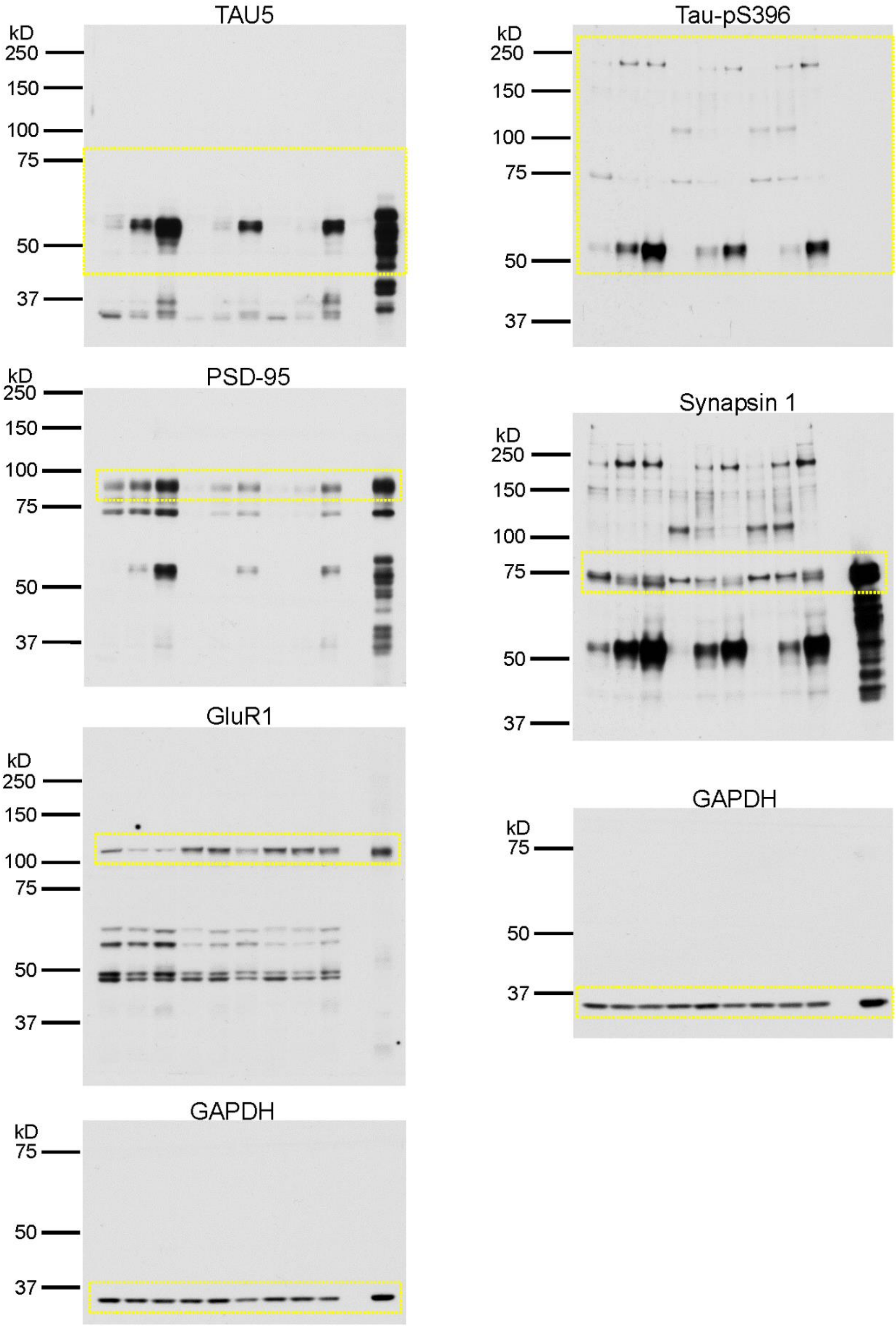

Supplementary Fig. S11. Full-length blots related to figures 2 and 3.

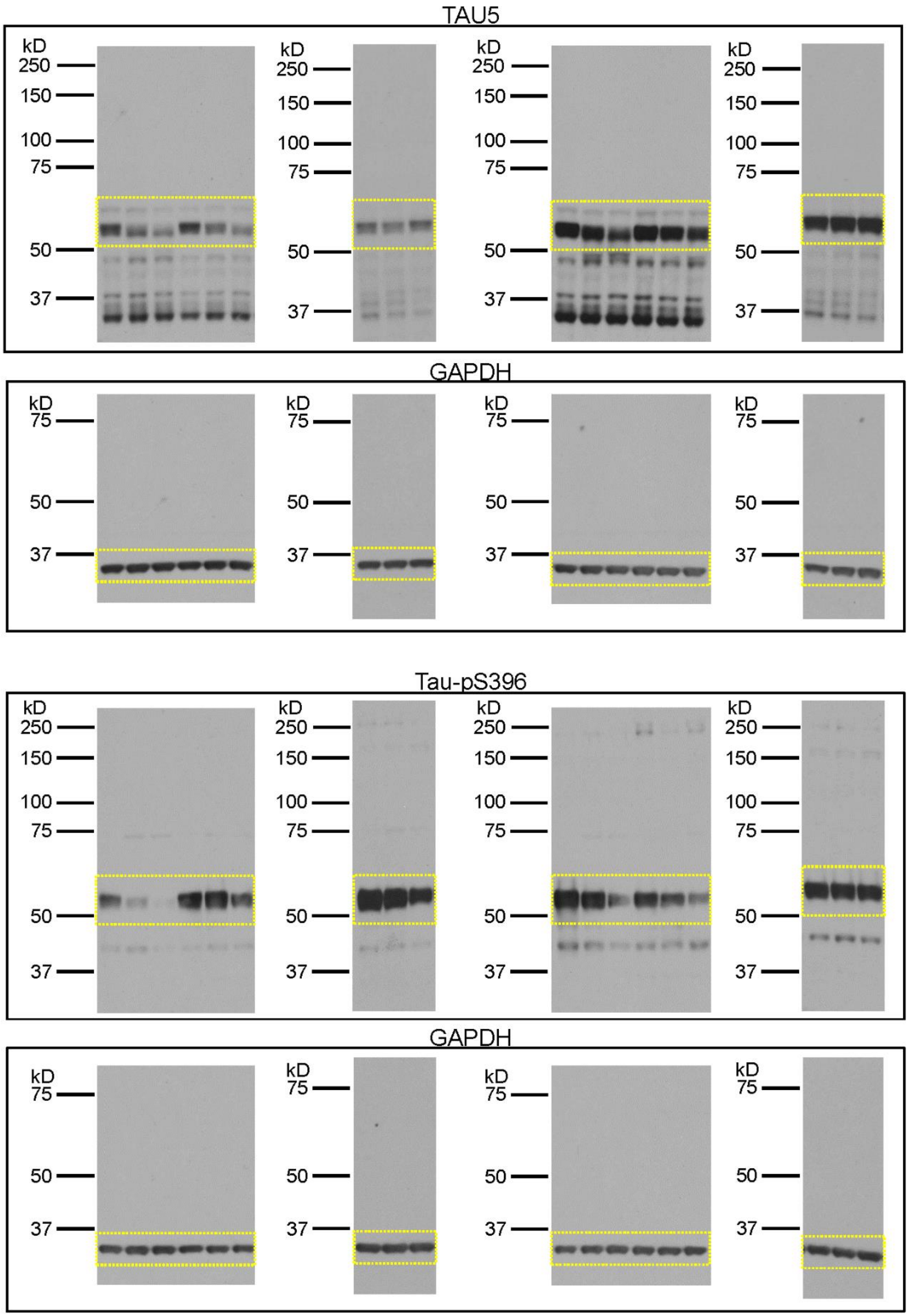

Supplementary Fig. S12. Full-length blots related to figure 7.
